# *SMPD4*-mediated sphingolipid metabolism regulates brain and primary cilia development

**DOI:** 10.1242/dev.202645

**Published:** 2024-11-15

**Authors:** Katherine A. Inskeep, Bryan Crase, Thamara Dayarathna, Rolf W. Stottmann

**Affiliations:** ^1^ Division of Developmental Biology, Cincinnati Children's Hospital Medical Center, Cincinnati, OH 45229, USA; ^2^ Steve and Cindy Rasmussen Institute for Genomic Medicine, Abigail Wexner Research Institute, Nationwide Children's Hospital, Columbus, OH 43205, USA; ^3^ Department of Neuroscience, The Ohio State University College of Arts and Sciences, Columbus, OH 43210, USA; ^4^ Department of Pediatrics, The Ohio State University College of Medicine, Columbus, OH 43210, USA

**Keywords:** Microcephaly, Cerebellar hypoplasia, SMPD4, Sphingolipids, Primary cilia, iPSCs, Mouse, Ceramide

## Abstract

Genetic variants in multiple sphingolipid biosynthesis genes cause human brain disorders. A recent study looked at people from 12 unrelated families with variants in the gene *SMPD4*, a neutral sphingomyelinase that metabolizes sphingomyelin into ceramide at an early stage of the biosynthesis pathway. These individuals have severe developmental brain malformations, including microcephaly and cerebellar hypoplasia. The disease mechanism of *SMPD4* was not known and so we pursued a new mouse model. We hypothesized that the role of *SMPD4* in producing ceramide is important for making primary cilia, a crucial organelle mediating cellular signaling. We found that the mouse model has cerebellar hypoplasia due to failure of Purkinje cell development. Human induced pluripotent stem cells lacking *SMPD4* exhibit neural progenitor cell death and have shortened primary cilia, which is rescued by adding exogenous ceramide. *SMPD4* production of ceramide is crucial for human brain development.

## Teaser

**Summary:** Mouse and human stem cell models of SMPD4 loss of function demonstrate that SMPD4 promotes cilia function and neural development.

## INTRODUCTION

The human brain is a uniquely complex structure. Neurological disorders represent a significant healthcare burden and are an area of intense research. The ability to sequence the human genome has greatly facilitated the study of genetic variants causing rare monogenic pediatric brain disorders. A recent cohort of 23 individuals from 12 families with sphingomyelin phosphodiesterase 4, neutral membrane (*SMPD4*; Magini et al., 2019) variants displayed phenotypes such as microcephaly cerebellar hypoplasia, delayed or hypomyelination of the brain, and severe developmental delay (7/7). Two additional pediatric individuals and three adult individuals with similar phenotypes, including insulin-dependent diabetes, have also been reported (Bijarnia-Mahay et al., 2022; Yamada et al., 2022; Smits et al., 2023).

Microcephaly, defined by a head circumference below three standard deviations, causes intellectual disability and developmental delay (Dobyns, 2002; Woods et al., 2005). In the USA, 1:800-5000 babies are born with microcephaly annually; or about 25,000 US births per year (Becerra-Solano et al., 2021). The development of the human cerebral cortex is a complex process with multiple steps including proliferation and differentiation of progenitor cells, migration of neuronal precursors into the cortical plate, and organization into the six layers of the mature cortex (Lee, 2019; Molnár and Clowry, 2012; Molnár et al., 2019). The balance between proliferative and asymmetric divisions of progenitor cells in the ventricular zone (VZ) is essential for generating the complex and expanded cerebral cortex of the human brain relative to other species. Many of the genes tied to primary microcephaly in humans are associated with the centrosome and mitotic spindle, which regulate cell division (Bond et al., 2002, 2005; Gilmore and Walsh, 2013; Guernsey et al., 2010; Jackson et al., 2002; Kumar et al., 2009). Similarly, defects in primary cilia-associated genes cause cell cycle and neural progenitor defects (Gilmore and Walsh, 2013; Jayaraman et al., 2016; Zhang et al., 2019). While there are species-specific differences between mouse and human corticogenesis, mouse models have generally served as a reliable model for these facets of cortical development (Capecchi and Pozner, 2015; Gruber et al., 2011; McIntyre et al., 2012; Putkey et al., 2002).

Cerebellar hypoplasia is characterized simply by smaller cerebellar size and global underdevelopment (Accogli et al., 2021). Cerebellar granule cells are the most abundant cell type in the mammalian brain. Unlike in the cerebral cortex, the cerebellar germinal zone is closest to the pial surface, and granule cells migrate internally after birth as the cerebellum develops (Altman and Bayer, 1985a; Fujita, 1967). The sonic hedgehog (SHH) pathway is crucial for cerebellar development (Wechsler-Reya and Scott, 1999). Purkinje cells are the only output neurons of the cerebellar cortex and are not produced from the external granule layer (EGL) but do secrete SHH required for granule cell proliferation (Butts et al., 2014; Corrales et al., 2004; Fujita, 1967; Seto et al., 2014). Granule cell progenitors (GCPs) and Purkinje cells are ciliated (Del Cerro and Snider, 1972; Doughty et al., 1998), and these primary cilia are required for their survival, integrity and localization (Bowie and Goetz, 2020). Accordingly, many ciliopathy disorders affect the cerebellum (Abdelhamed et al., 2013; Epting et al., 2020; Inskeep et al., 2022; Lee and Gleeson, 2011). Cortical and cerebellar development have both similarities and intriguing differences that become relevant in our study.

*SMPD4* is a member of the sphingomyelinase family, which hydrolyzes sphingomyelin to produce ceramide and phosphorylcholine in the catabolic pathway of sphingolipid biosynthesis (Krut et al., 2006). Ceramide is the precursor of most complex sphingolipids. Disorders of sphingolipid content include Krabbe's disease, Gaucher's disease and Niemann-Pick disease (*SMPD1*) (Platt, 2014). These lipid storage disorders all involve improper accumulation of sphingolipids or lipid components in the lysosome. Ceramide is a cornerstone lipid with diverse roles in the cell. Studies have linked cell stress and apoptosis induction to increased ceramide levels (Herget et al., 2000; Kolesnick and Krönke, 1998; Lee et al., 2015). However, its roles in cell division and primary ciliogenesis are of particular interest to our study. Ceramide interacts with acetylated tubulin to stabilize centrosome-associated microtubules (Tripathi et al., 2021). It is well known that ceramide is required for primary ciliogenesis, and specifically in neural progenitor cells (He et al., 2014; Wang et al., 2009). Primary cilia and centrosome cycling are inextricably linked to the cell cycle (Kim and Tsiokas, 2011).

In this study, we sought to establish a mouse model and study the molecular mechanism of *SMPD4*-mediated structural brain malformations. First, we determined the developmental timeline of *Smpd4* expression in the mouse brain. We found that *Smpd4* is highly expressed in developing forebrain, particularly in the VZ, starting at embryonic day (E)12.5, and in both granule and Purkinje cells in the cerebellum from E14.5. We present an *Smpd4* null mouse model with incompletely penetrant perinatal lethality, failure to thrive and cerebellar hypoplasia. We further determined that the cause of cerebellar defect is likely due to loss of Purkinje cells. *Smpd4* null and forebrain-specific knockout mice do not exhibit the microcephaly seen in human patients. We established human stem cell models which showed a loss of neural progenitor cells due to cell death and decreased proliferation. We explored the role of primary cilia in these models and found that, although *Smpd4* null mice appear to be unaffected, human induced pluripotent stem cell (iPSC) models have shortened and bulbous primary cilia. We propose that this is due to changes in subcellular ceramide, as treating iPSCs with exogenous ceramide significantly rescues cilia length. We also found evidence for disrupted WNT pathway signaling. We suggest that during brain development, *SMPD4* provides ceramide in an organelle-specific manner, regulating the cell cycle and allowing primary cilia to grow properly.

## RESULTS

### Smpd4 is highly expressed in the developing mouse brain

A comprehensive analysis of *Smpd4* expression in the developing mouse brain has not been previously carried out. Human *SMPD4* is expressed in most tissue types throughout embryonic and postnatal development including brain and human-derived neural organoids (Fietz et al., 2012; Luo et al., 2016; Zhu et al., 2016). Similar RNA-seq experiments in mouse show *Smpd4* expression in multiple brain regions including the VZ, subventricular zone (SVZ) and cortical plate (Fietz et al., 2012), but without detailing forebrain or cerebellum expression.

**Fig. 1.**
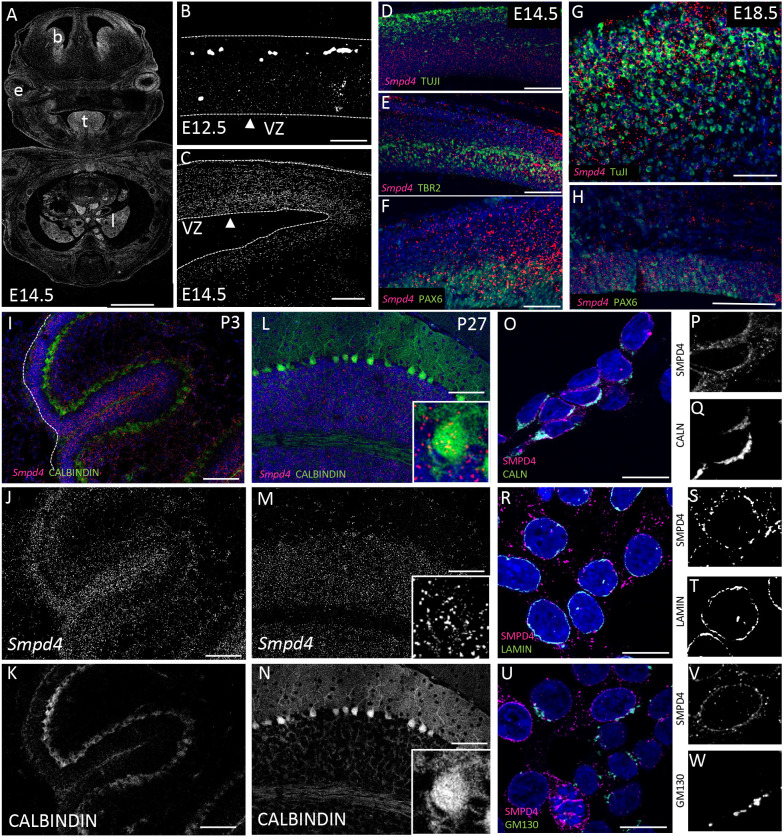
***Smpd4* is expressed throughout mouse cortical and cerebellar development.** (A) *Smpd4* expression in mouse embryo at E14.5 shows *Smpd4* expression in the brain (b), eyes (e), tongue (t) and lungs (l). (B,C) Expression in the forebrain is shown at E12.5 (B) and E14.5 (C). Ventricular surface is marked with an arrowhead. VZ, ventricular zone. (D-H) Serial sections of *Smpd4* RNA expression and TuJI (D,G), TBR2 (E) and PAX6 (F,H) immunohistochemistry show that *Smpd4* colocalizes with multiple cell types at E14.5 (D-F) and E18.5 (G,H). (I-N) *Smpd4* expression in mouse cerebellum at P3 (I-K) and P27 (L-N) is in both the external (P3) and internal (P27) granule cell layers and calbindin^+^ Purkinje cells. White dashed line (I) indicates the edge of the cerebellum. Insets (L-N) show a higher magnification of one individual Purkinje cell body. (O-W) SMPD4 does colocalize with calnexin in the ER (O-Q) and lamin^+^ nuclear membrane (R-T), but not GM130 in the Golgi apparatus (U-W). Scale bars: 1 mm (A); 100 μm (B-H); 200 μm (I-N); 20 μm (O,R,U).

We assayed *Smpd4* mRNA forebrain expression via RNAScope probes from E12.5 to postnatal day (P)27. Expression is highest at E14.5, the peak of neurogenesis in the mouse cortex corresponding to the end of the second trimester in human development (Chen et al., 2017). *Smpd4* is widely expressed at E14.5, particularly in the brain, lungs, tongue and eye (Fig. 1A). We observed forebrain expression starting at E12.5 (Fig. 1B) and continuing throughout development. At E14.5, *Smpd4* continues to be strongly expressed near the ventricle (Fig. 1C) but is co-expressed with TuJI in differentiated neurons (Fig. 1D), TBR2 and intermediate progenitors (Fig. 1E) as well as PAX6 and neural progenitor cells in the VZ (Fig. 1F) (Estivill-Torrus et al., 2002; Heins et al., 2002). All of these domains are consistent with previous large datasets describing embryonic expression (Diez-Roux et al., 2011). We found that *Smpd4* is gradually enriched in the VZ by E18.5 (Fig. 1H) relative to the cortical plate (Fig. 1G) but is maintained at low levels across the cortex. These data show that *Smpd4* is expressed at crucial stages of neurodevelopment, consistent with widespread expression in previous data (Lein et al., 2007; Uhlen et al., 2015; Zhang et al., 2014).

Additionally, we observed widespread *Smpd4* expression at very early stages in the cerebellar primordium at E14.5 (Fig. 1I-K). Expression is maintained to maturation at P27 (Fig. 1L-N). *Smpd4* is expressed in multiple cell types of the cerebellum, most highly in granule cells of both the external layer at P3 and the internal layer at P27 but also in calbindin^+^ Purkinje cells (Fig. 1I-N). At the organelle level, SMPD4 localizes to both the calnexin^+^ endoplasmic reticulum (ER) (Fig. 1O-Q) and lamin^+^ nuclear membrane (Fig. 1R-T), but not the GM130^+^ Golgi apparatus (Fig. 1U-W), unlike previously reported (Stoffel et al., 2016). We have detailed the expression of *Smpd4* in neurogenesis and found it is consistent with a role in both cortical and cerebellar development.

### Loss of Smpd4 causes perinatal demise, failure to thrive and cerebellar hypoplasia

We hypothesized that *Smpd4*-null mice would exhibit structural brain malformations consistent with human phenotypes. The International Mouse Phenotyping Consortium generated a conditional gene trap allele of *Smpd4* [*Smpd4^tm2a(KOMP)Wtsi^*], and *Smpd4^tm2b^* (i.e. *Smpd4^null^*) mice exhibited incompletely penetrant lethality at weaning but noted no further developmental abnormalities. We used this line to determine the role of *Smpd4* in embryonic and postnatal brain development.

**Fig. 2.**
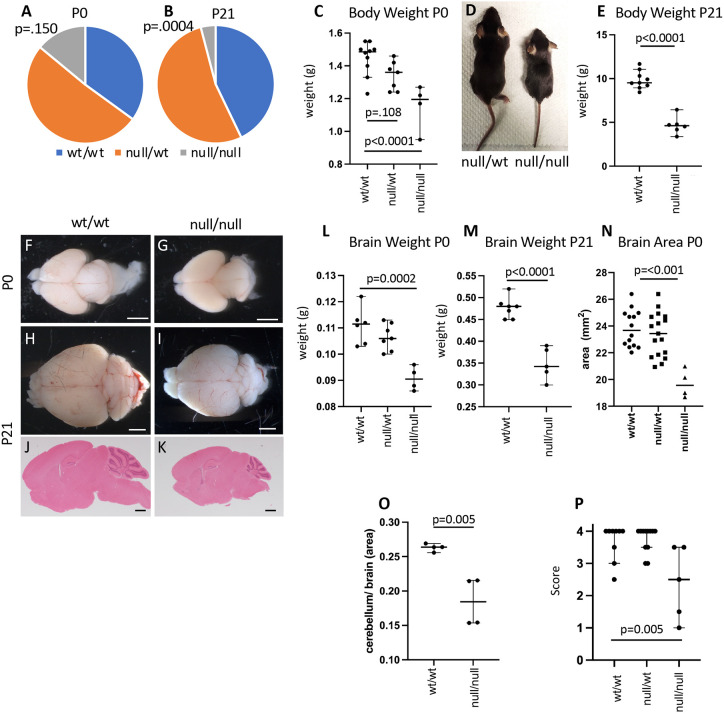
**Loss of *Smpd4* causes perinatal demise, cerebellar hypoplasia and microcephaly in surviving animals.** (A,B) *Smpd4* homozygous null animals survive at sub-Mendelian ratios after birth. (C-E) They weigh significantly less than their littermates at P0 (C; *n*=10 wt, 7 null/wt, 4 null/null; ANOVA, *P*=0.0006) and weaning (D,E; *n*=9 wt, 6 null/null; *P*<0.0001). (F-P) Brains dissected from null animals look largely normal (F-K) but weigh less at P0 (L; *n*=6 wt, 7 null/wt, 4 null/null; ANOVA, *P*=0.0003) and P21 (M; *n*=6 wt, 4 null/null; *P*<0.0001). The homozygous null brains are microcephalic with smaller dorsal surface area at P0 (N; *n*=14 wt, 17 null/wt, 4 null/null; ANOVA, *P*<0.0001) and cerebellar hypoplasia at P21 (O; *n*=3 wt, 4 null/null; *P*=0.005). At P21, homozygous null animals exhibit hindlimb clasping indicating a cerebellar deficit (P; *n*=9 wt, 12 null/wt, 5 null/null; ANOVA, *P*=0.0017). Data are median±95% confidence interval. Scale bars: 2 mm (F-I); 1 mm (J,K).

Almost half of the *Smpd4^null/null^* mice died before P0 and surviving animals were significantly smaller at P0 and weaning (Fig. 2A-E). Only 4% survived to weaning (*n*=7/23 expected, *P*=0.004; Fig. 2B; Table S4). Surviving animals all failed to thrive (Fig. 2D), were about half the weight of their littermates (Fig. 2E), had no sex bias, and subsequently required euthanasia. *Smpd4^null/null^* animals had brains that looked grossly normal (Fig. 2F-K) but were significantly smaller as measured by weight at P0 (Fig. 2L) and P21 (Fig. 2M) and had a smaller dorsal surface area at P0 (Fig. 2N). Molecular analyses failed to identify a specific molecular cause for reduced brain size (Fig. S1) and there was no evidence of disproportionately small forebrains in mutants.

However, surviving *Smpd4^null/null^* mice have severe cerebellar hypoplasia (Fig. 2O). Surviving animals showed hindlimb clasping consistent with cerebellar deficits (Fig. 2P). There was no statistical difference in body or brain weight between P21 males and females (*P*=0.991). Overall, these brain and behavior phenotypes are partially consistent with those observed in published human *SMPD4* patients (Magini et al., 2019).

### Purkinje cells fail to support cerebellar postnatal development in the absence of Smpd4

To circumvent the apparent early postnatal lethality of the *Smpd4* germline model, we decided to employ a conditional genetic strategy. We used the Cre-lox system, in which loxP sites surrounding a crucial exon in a gene of interest (i.e. ‘floxed’) can be combined with tissue-specific Cre recombinase-expressing transgenic mice. This affords specific spatiotemporal control in deleting the gene of interest. We used Cre transgenic lines to specifically delete *Smpd4* in three discrete lineages: the hindbrain (*Engrailed1-*Cre; *En1*-Cre), dorsal telencephalon (*Emx1-*Cre) and forebrain (*Foxg1-*Cre).

**Fig. 3.**
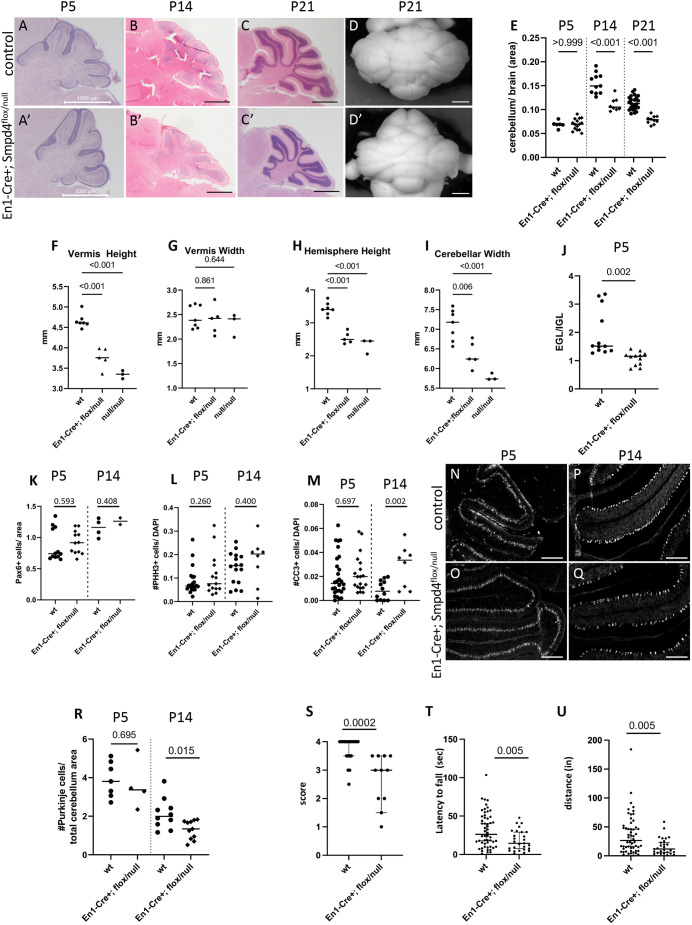
***Smpd4* is required for proper Purkinje cell support of postnatal cerebellar development.** (A-D′) *En1*-Cre mediated *Smpd4* deletion leads to a progressively underdeveloped cerebellum. (E,J) Cerebellar area relative to brain area is not yet reduced at P5 (E; *n*=6 wt, 15 conditional knockout; ANOVA, *P*>0.999) but is smaller at P14 (*n*=11 wt, 8 conditional knockout; ANOVA, *P*<0.001) and P21 (*n*=17 wt, 15 conditional knockout; ANOVA, *P*<0.001) and shows premature exit of granule precursor cells from the external to internal granule layer at P5 (J; *n*=12 wt, 12 conditional knockout; *P*=0.002). (F-I) By P21, the height of the vermis (F; *n*=7 wt, 5 conditional knockout, 3 homozygous null; ANOVA, *P*<0.001) and hemispheres (H; *n*=7 wt, 5 conditional knockout, 3 homozygous null; ANOVA, *P*<0.001) and the overall width of the cerebellum (I; *n*=7 wt, 5 conditional knockout, 3 homozygous null; ANOVA, *P*<0.001) are drastically reduced, although vermian width appears to be unaffected (*n*=7 wt, 5 conditional knockout, 3 homozygous null; ANOVA, *P*=0.064; G). (K-M) The number of granule precursor cells in the external granule layer marked by PAX6 (K; *n*=12 wt, 12 conditional knockout, *P*=0.593 at P5; *n*=4 wt, 2 conditional knockout, *P*=0.408 at P14) and cell division rates as measured by pHH3 (L; *n*=22 wt, 15 conditional knockout, *P*=0.260 at P5; *n*=16 wt, 8 conditional knockout, *P*=0.400 at P14) are not different at P5 and P14. Cell death (M; CC3, *n*=28 wt, 19 conditional knockout, *P*=0.697 at P5; *n*=12 wt, 8 conditional knockout, *P*=0. 002 at P14) is increased at P14 but not P5. (N-R) A specific loss of Purkinje cells is seen between P5 and P14 (*n*=7 wt, 4 conditional knockout, *P*=0.695 at P5; *n*=10 wt, 10 conditional knockout, *P*=0.015 at P14). (S-U) The conditional deletion mice have corresponding cerebellar behavioral phenotypes including hindlimb clasping (S; *n*=20 wt and 11 conditional knockout, *P*=0.0002) and decreased latency to fall (T; *n*=54 wt and 27 mutant, *P*=0.0051) and distance traveled on a rotarod (U; *n*=54 wt and 27 mutant, *P*=0.0046). Data are median±95% confidence interval. Scale bars: 1 mm (A-D′); 200 μm (N-Q).

Motivated by cerebellar hypoplasia in *SMPD4* patients and the *Smpd4* germline model, we generated a hindbrain-specific deletion of *Smpd4* using *En1*-Cre. *En1-Cre^+^; Smpd4^flox/null^* animals survived to weaning (Table S5) but were smaller and ataxic compared to their littermates. While the cerebellum was morphologically normal in early postnatal cerebellar development (P5, Fig. 3A), it appeared to be obviously smaller at P14 (Fig. 3B). P21 *En1-Cre+; Smpd4^flox/null^* animals had readily apparent cerebellar hypoplasia (Fig. 3C,D, quantified in Fig. 3E). Specifically, they had reduced vermian height, hemisphere width and overall cerebellar width, without reduced vermian width (Fig. 3F-J). We also showed these same features of altered cerebellar development in three *Smpd4^null/null^* animals that survived to this stage (Fig. 3F-J).

Next, we assessed which cell type(s) are perturbed leading to the smaller cerebellar size and quantified the number of cells in both the EGL and internal granule layer (IGL). *En1-Cre^+^; Smpd4^flox/null^* animals had a lower ratio of EGL to IGL cells at P5, suggesting that more granule precursor cells were already exiting the proliferative EGL and populating the nascent IGL (Fig. 3J). We hypothesized that this is due to premature differentiation and/or migration of the granule precursor cells. We quantified these PAX6^+^ cells and found no difference in their number between control and *En1-Cre*^+^*; Smpd4^flox/null^* animals at P5 or P14 (Fig. 3K). Similarly, pHH3^+^ proliferative cells were not reduced (Fig. 3L). We did find an increase in apoptotic cells (CC3^+^) at P14, but not at P5 (Fig. 3M; Fig. S2). We noted that most of the dying cells were in the Purkinje cell layer. While calbindin^+^ Purkinje cells were present in normal numbers at P5, they fail to survive and are markedly decreased at P14 (Fig. 3N-Q, quantified in Fig. 3R).

Purkinje cell loss often causes ataxic behaviors. We scored P21 *En1-Cre^+^; Smpd4^flox/null^* mice for behavior indicating cerebellar ataxia (Lukacs et al., 2020) and found that they exhibit hindlimb clasping (Fig. 3S). We also tested them at 6 weeks of age on a rotarod to further quantify cerebellar ataxia (Deacon, 2013). As expected, *En1-Cre^+^; Smpd4^flox/null^* mice performed worse than their littermates, with shorter latency to fall and shorter total distance traveled on the rod (Fig. 3T,U). No sex bias was observed with either behavioral test. Overall, these data support a model wherein *Smpd4* is required for postnatal survival, but not initial generation, of Purkinje cells. Purkinje cell loss results in a smaller cerebellum and ataxic behavior in the mice.

### Forebrain-specific deletion of Smpd4 does not cause microcephaly

*Emx1* is expressed in the forebrain lineage as early as E10.5 (Gorski et al., 2002). We hypothesized that such a specific deletion may reveal a microcephaly phenotype given the patient phenotypes, while circumventing lethality. *Emx1-Cre^+^; Smpd4^flox/null^* mice survive in Mendelian ratios to weaning but are not microcephalic (Table S6; Fig. S3A-J). To test the idea that this ablation may not have been early enough in forebrain development, we next used *Foxg1*-*Cre*, which is expressed in the telencephalic lineage beginning at E8.75 (Hébert and McConnell, 2000; Kawaguchi et al., 2016). *Foxg1-Cre^+^; Smpd4^flox/null^* mice likewise survive to weaning (Table S7) and, also, do not exhibit microcephaly (Fig. S3K-R). Because humans with *SMPD4* deficiency have microcephaly, we hypothesized there is either a compensatory mechanism in mouse, or that human neurodevelopmental differences explain this disparity.

### Neutral sphingomyelinase genes do not compensate for each other in brain development

Given our finding that conditional *Smpd4* loss in the mouse forebrain does not lead to microcephaly as in humans, we hypothesized that neutral sphingomyelinase genes could act redundantly in the mouse. *Smpd3* and *Smpd4* are the two family members with highest expression in the developing brain (Fietz et al., 2012; Krut et al., 2006). We further detailed *Smpd3* mRNA expression across forebrain and cerebellar development (E12.5-P21). *Smpd3* is restricted to upper cortical layers throughout forebrain development at E14.5 and E18.5 (Fig. S4A,B). It is also expressed in the cerebellar primordium (Fig. S4C,D). We observed no upregulation in mRNA expression of either neutral sphingomyelinase in the absence of the other (Fig. S4E-P). SMPD3 protein was previously shown to localize to the Golgi apparatus (Stoffel et al., 2005), the ER and the cell membrane (Piët et al., 2022). We did not observe localization of SMPD3 to the GM130^+^ Golgi apparatus (Fig. S4Q) or lamin^+^ nuclear membrane (Fig. S4R). SMPD3 is localized to the calnexin^+^ ER (Fig. S4S). Our RNA expression and protein subcellular localization data show some, but not complete, overlap of SMPD3 and SMPD4. We therefore genetically tested the hypothesis that there may be some functional redundancy between *Smpd3* and *Smpd4* during CNS development.

Previous studies have shown that *Smpd3* deletion in mouse models causes juvenile dwarfism, delayed puberty, skeletal growth inhibition due to disruption of the Golgi secretory pathway of chondrocytes and progressive cognitive impairment similar to Alzheimer's disease (Stoffel et al., 2016, 2019). This allele of *Smpd3* was made on a C57BL/6x129Sv genetic background and analysis was reported as a congenic C57BL/6 (B6) line. The independently derived *fragilitus ossium Smpd3* allele recovered from a forward genetic screen has osteogenesis imperfecta (‘brittle bone’ disease), shortened and bent long bones, tooth development issues and partially penetrant postnatal lethality. We were unable to obtain either of these alleles and therefore generated a novel *Smpd3* deletion allele on a B6 background (Fig. S5A,B, hereafter referred to as *Smpd3^del^*). We found that deletion of *Smpd3* causes perinatal lethality, as we never recovered any live *Smpd3^del/del^* mice at birth (P0) (Fig. S5C; Table S8). *Smpd3^del^*^/del^ E18.5 embryos have severe skeletal abnormalities (Fig. S5D-G) including shortened mandibles (Fig. S5H) and long bones (radius, humerus and scapula; Fig. S5I-K). However, we did not note any skeletal abnormalities in E18.5 *Smpd4^null/null^* embryos (Fig. S6). *Smpd3^del/del^* embryos do not exhibit any obvious cortical abnormalities at E14.5 or E16.5 (Fig. S5L-N). On a CD1 background, *Smpd3^del/del^* mice likewise exhibit complete perinatal lethality (Table S9) and similar severe skeletal abnormalities (Fig. S5O-V). Given the earlier lethality in this model compared to previous studies, we performed whole-genome sequencing of an *Smpd3^del/del^* homozygote to ensure the fidelity of our allele. We found the genomic deletion was indeed specific to *Smpd3* and there were no other appreciable deletions on chromosome 8 (Fig. S7). Our data show that *Smpd3* is required for postnatal survival and skeletal development but loss of *Smpd3* alone does not lead to any dramatic cortical malformations.

Accordingly, *Smpd3^del/del^; Smpd4^null/null^* double knockout (dKO) mice do not survive birth and we noted some lethality at late embryonic stages (*n*=8 of 19.75 expected at E16.5-E18.5; *P*=0.001; Table S10). However, we did not observe any embryonic structural cortical abnormalities in dKO animals (Fig. S4T-U′). Overall, we saw no evidence for functional compensation in mouse brain development between these two neutral sphingomyelinase genes.

### Human *SMPD4*-deficient iPSC models exhibit cell death and decreased proliferation

**Fig. 4.**
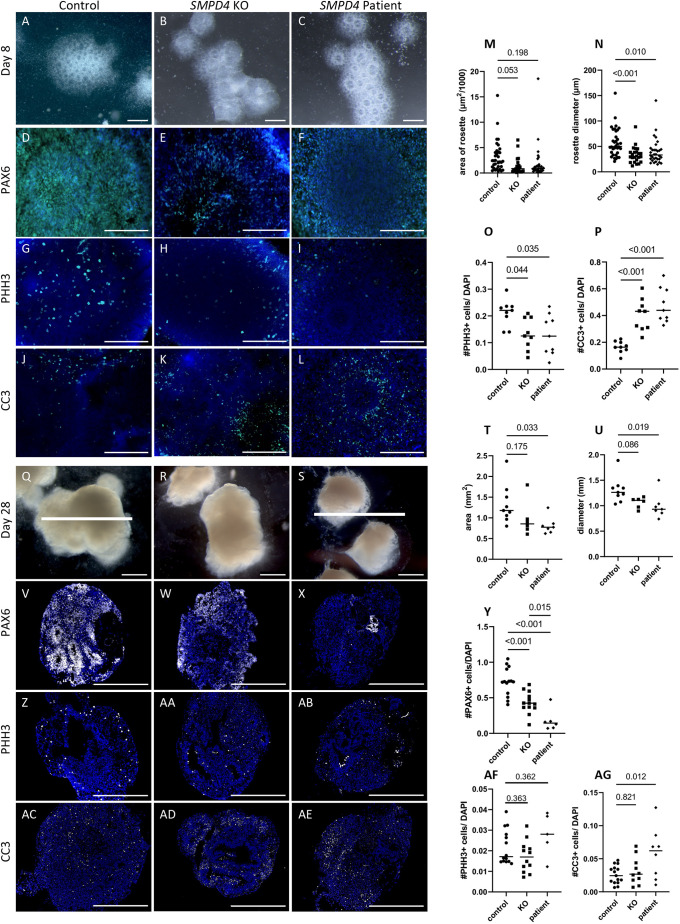
**Human iPSC models show reduced neural progenitor cells, decreased proliferation and increased cell death.** (A-P) Neural rosettes generated from control iPSCs at 8 days in culture show normal rosette formation (A), whereas *SMPD4* knockout (B) and patient (C) rosettes are structurally abnormal and smaller in both area (M; *n*=46 control, 24 knockout, 32 patient; ANOVA, *P*=0.027) and diameter (N; *n*=46 control, 24 knockout, 32 patient, ANOVA *P*<0.0001). Immunohistochemistry for neural progenitors (D-F; PAX6), cell proliferation (G-I,O; pHH3, *n*=9 control, 9 knockout, 9 patient; ANOVA, *P*=0.001) and cell death (J-L,P; CC3, *n*=9 control, 9 knockout, 9 patient; ANOVA, *P*=0.0004) indicating a decrease in proliferative cells and increase in apoptotic cells in both *SMPD4* knockout and patient rosettes. (Q-AG) Neural organoids generated from iPSCs likewise show that knockout and patient organoids are smaller than control (Q-U; *n*=9 control, 5 knockout, 7 patient; area: ANOVA, *P*=0.046; diameter: ANOVA, *P*=0.0232). Knockout and patient organoids have a distinct loss of PAX6^+^ progenitor cells (V-Y; *n*=14 control, 13 knockout, 6 patient; ANOVA, *P*<0.0001). The number of pHH3^+^ dividing cells is unchanged (Z-AB,AF; *n*=14 control, 12 knockout, 5 patient; ANOVA, *P*=0.069), but apoptosis is increased in patient organoids (AC-AE,AG; *n*=15 control, 9 knockout, 8 patient; ANOVA, *P*=0.014). Data are median. Scale bars: 200 µm (D-L); 500 µm (Q-AE). White bar in Q and S are the same length to highlight smaller size of patient organoids.

Mouse neurogenesis is a model for many aspects of human neurogenesis, but the human forebrain is markedly more complex. Therefore, we hypothesized that a human model might reveal specific consequences for cortical neurogenesis upon loss of *SMPD4*. We acquired fibroblasts from an individual with *SMPD4* deficiency and reprogrammed them into iPSCs. We also created an *SMPD4* knockout iPSC line (*SMPD4* KO) using CRISPR/Cas9 as we were unable to correct the *SMPD4* variant in the human-derived cells to create a true isogenic control (Fig. S8). Quality control experiments were performed on these cell lines and confirmed relative identities through short tandem repeat profiling, showed that the cells retained the capacity to generate all three cell lineages and had largely normal karyotypes (Fig. S9). Neural rosettes are two-dimensional structures of neural progenitor cells (NPCs) with radial organization similar to the *in vivo* cortex (Fedorova et al., 2019; Knight et al., 2018; Zhang et al., 2001). We generated neural rosettes from these iPSCs and found several abnormalities in both patient and KO rosettes. Brightfield images after 8 days in culture show that rosettes from individuals with *SMPD4* deficiency and KO rosettes exhibited disrupted morphology, with cells missing from the center (Fig. 4A-C). Specifically, they have a smaller area and diameter (Fig. 4M,N), fewer PAX6^+^ neural progenitors (Fig. 4D-F), a 50% reduction in proliferation levels (pHH3, *P*<0.05; Fig. 4G-I,O) and a two-fold increase in CC3^+^ apoptotic cells (*P*<0.001; Fig. 4J-L,P).

While neural rosettes are a good two-dimensional model for early neurogenesis, neural organoids permit three-dimensional study of human brain development at later stages *in vitro* (Lancaster et al., 2013). After 28 days *in vitro*, neural organoids generated from iPSCs from individuals with *SMPD4* deficiency and KO iPSCs were smaller than control organoids (Fig. 4Q-U). Interestingly, we detected an even more severe loss of PAX6^+^ NPCs in the organoid model compared to the neural rosettes (Fig. 4V-Y). Proliferation levels were similar (Fig. 4Z-AB,AF) and cell death was less severe than in the neural rosettes, although still increased in patient-derived organoids (Fig. 4AC-AE,AG). We hypothesize that the proliferation and cell death differences between these two iPSC models is because they model different stages of cortical development. An early loss of NPCs as seen in the neural rosette model disrupts early neurogenesis, explaining a more severe size difference persisting in the organoid model of later stage development.

Premature loss of NPCs, decreased proliferation and increased cell death are common molecular mechanisms of human microcephaly (Insolera et al., 2014; Jayaraman et al., 2016; Kaindl et al., 2010; Kumar et al., 2009; McIntyre et al., 2012). Because fewer NPCs are present, proliferate less and fail to survive, many fewer total neurons are produced. We propose a combination of these mechanisms to explain the microcephaly seen in individuals with *SMPD4* deficiency. We hypothesize that the difference between our mouse and human iPSC models indicates a species-specific use of *SMPD4* in cortical development.

### SMPD4 loss leads to cell-specific primary cilia defects

**Fig. 5.**
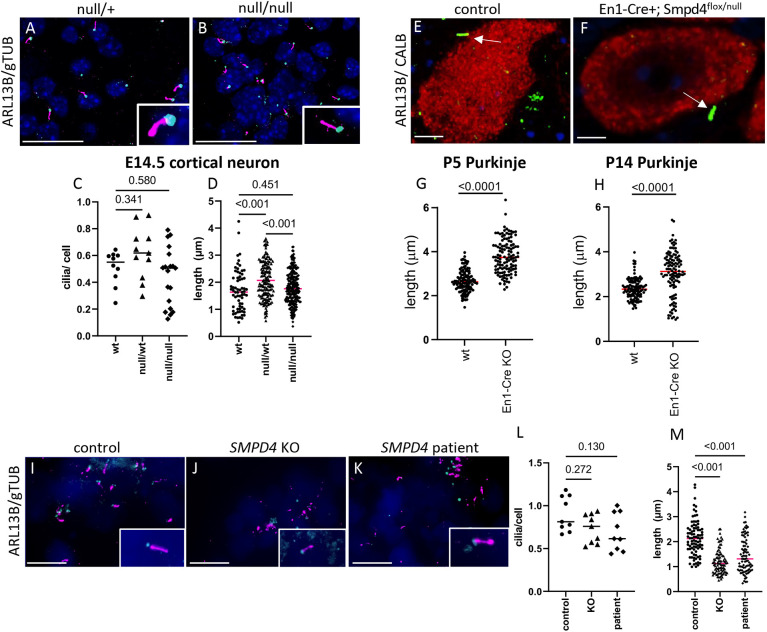
**Primary cilia are disrupted by *SMPD4* loss.** (A,B) Primary cilia in neurons cultured from heterozygous *Smpd4^null/wt^* and homozygous *Smpd4^null/null^* E14.5 cortex are highlighted by ARL13B (purple) in the ciliary membrane and γ-tubulin (gTUB; blue) at the basal body. (C,D) Cilia appear to be unaffected in number (C; *n*=11 wt, 11 null/wt and 19 null/null) or length (D; *n*=71 wt, 80 null/wt and 115 null/null; ANOVA, *P*=0.0093) between wt and null/null. (E-H) Tissue sections of postnatal mouse cerebellum stained with ARL13B (green) and calbindin (red; E,F) show that, at P5 and P14, the primary cilia in *En1-Cre^+^; Smpd4^flox/null^* animals are longer (G,H; *n*=118 cilia at each age and genotype; *P*<0.001). Arrows show individual cilia. (I-M) Representative images (I-K) of cilia (ARL13B in purple, gTUB in blue) in human iPSC-derived neural rosettes are present in normal numbers (L; *n*=9 control, 9 knockout and 9 patient; ANOVA, *P*=0.0768) but are significantly shorter (M; *n*=87 control, 87 knockout and 87 patient; ANOVA, *P*<0.0001; M) in *SMPD4* KO and patient. Insets (A,B,I-K) show individual cilia at higher magnification. Data are median. Scale bars: 10 µm (A,B,I,J,K); 3.3 µm (E,F).

Ceramide depletion has previously been shown to disrupt primary ciliogenesis (Wang et al., 2009) and cilia are crucial for normal brain development (Lee and Gleeson, 2011; Park et al., 2019). We hypothesized that loss of SMPD4 would decrease ceramide availability at the primary cilium. The primary cilia in cultured mouse E14.5 forebrain neurons from wild-type (WT) and homozygous *Smpd4^null/null^* all appeared to be comparable in number and length, although we note an apparent slight increase in length in heterozygous *Smpd4^null/wt^* neurons (Fig. 5A-D). Because we have shown that the *En1-Cre^+^; Smpd4^flox/null^* mouse cerebellar defect is due to Purkinje cell death, we also looked specifically at these cells. Interestingly, while the number of cilia was unaffected, *En1-Cre^+^; Smpd4^flox/null^* Purkinje cell cilia were, on average, longer at both postnatal stages we assayed (P5 and P14; Fig. 5E-H).

Cilia in human *SMPD4* KO and patient iPSC neural rosettes were found in normal numbers but are shortened and sometimes dysmorphic (control length=2.13 µm, KO=1.19 µm, patient=1.45 µm, *P*<0.001; Fig. 5I-M). *SMPD4* KO and patient organoids also had shortened, dysmorphic primary cilia without a significant decrease in number (Fig. S10). The observed dysmorphisms were not uniform, but we noticed that some KO and patient rosette cilia had bulbous distal tips (insets in Fig. 5I-K).

### Loss of *Smpd4* does not lead to an accumulation of total sphingomyelin or decline in total ceramide in mouse brain

Neutral sphingomyelinases function in the recycling portion of the sphingolipid biosynthesis pathway to produce ceramide and phosphocholine from sphingomyelinase. The cell can use either this ‘recycling’ pathway or *de novo* synthesis, but the majority primarily use the recycling pathway (Gillard et al., 1998). Thus, we hypothesized that loss of SMPD4 sphingomyelinase activity will cause a sphingomyelin buildup and/or ceramide decrease in the cell, which could explain SMPD4 phenotypes. To test this, we performed mass spectrometry for sphingomyelin and ceramide species of *Smpd4* control and homozygous null E18.5 mouse brains in triplicate. Surprisingly, we found no overall change in sphingomyelin or ceramide in the cortex or cerebellum of mouse brain tissue (Fig. S11). These results are similar to those seen in *Smpd2* and *Smpd3* KO mice, which also do not exhibit a change in sphingomyelin levels (Stoffel et al., 2005). Numerous studies have suggested that the activity of sphingomyelinases is highly specific to the organelle(s) in which they are localized. Therefore, perturbation of one sphingomyelinase may lead to organelle-specific changes in sphingolipid content rather than a tissue- or even cell-level change, and thus be undetectable by this assay (Airola and Hannun, 2013; Hannun and Obeid, 2018).

### SMPD4 cilia defect is rescued by exogenous ceramide treatment

**Fig. 6.**
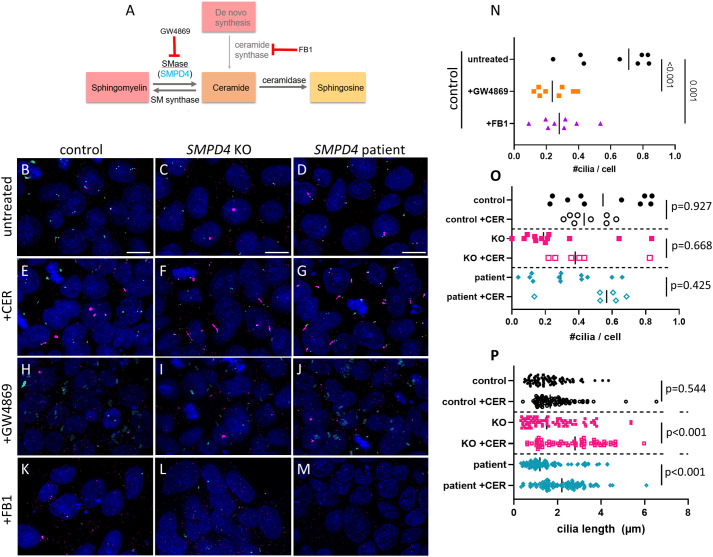
**Cilia shortening in *SMPD4* models is rescued by *in vitro* ceramide treatment.** (A) Schematic of ceramide synthesis and where compounds used in this study will act. (B-P) iPSCs were treated with PBS, C16 ceramide (CER), GW4869 or FB1. Treatment with GW4869 or FB1 decreases number of cilia in control iPSCs (N; *n*=8 each control, GW4869 or FB1; ANOVA, *P*<0.001). Treatment of *SMPD4* KO and patient iPSCs with ceramide does not impact the number (O; *n*=10 control, 8 ceramide treated, *P*=0.927; 12 knockout, 6 ceramide treated, *P*=0.668; 12 patient, 6 ceramide treated, *P*=0.425), but does increase the length of primary cilia per cell (P; *n*=21 control, 22 ceramide treated, *P*=0.544; 19 knockout, 30 ceramide treated, *P*<0.001; and 29 patient, 22 ceramide treated, *P*<0.001). Vertical bar (N-P) shows median. Scale bars: 20 µm (B-D, for B-M).

To test the effect of ceramide on promoting cilia growth, we treated human iPSC lines with exogenous C16 ceramide and inhibitors of ceramide synthesis (Fig. 6A-M). GW4869 is a neutral sphingomyelinase inhibitor (Luberto et al., 2002). FB1 is an inhibitor of the *de novo* ceramide synthesis pathway (Fukuda et al., 1996). We hypothesized that treatment of control iPSCs with either GW4869 or FB1 would deplete ceramide and thus decrease cilia number and/or length. Treatment of WT cells with GW4869 or FB1 did indeed result in fewer cilia in control iPSCs (Fig. 6N), confirming that ceramide depletion causes a cilia defect. Interestingly, GW4869 has no effect on KO or patient cells, as we would expect for cells that have already lost SMPD4 function given its known role as a neutral sphingomyelinase inhibitor (Luberto et al., 2002) (Fig. S12). *SMPD4* KO and patient cells do not have fewer cilia than control cells, and supplementation with ceramide did not significantly affect the number of primary cilia (*P*=0.668 and *P*=0.225, respectively; Fig. 6O). However, ceramide supplementation dramatically increased cilia length in *SMPD4* KO and patient cells (1.61 µm to 2.32 µm for patient, 1.43 µm to 2.19 µm for KO, *P*<0.001; Fig. 6P). We therefore conclude that loss of functional SMPD4 may lead to changes in local ceramide availability at the cilia, resulting in shortened and presumably dysfunctional cilia. We further tested this idea by treating cells with an HDAC inhibitor which has previously been shown to lengthen cilia (Shi et al., 2021; Xu et al., 2018), hypothesizing that rescuing cilia length may restore the proliferation deficit. We supplemented cultured iPSCs with ACY1215 and found that the treatment led to longer cilia in control cells but not in KO or patient cells (Fig. S13).

### SMPD4 may impact molecular signaling pathways

Primary cilia are known to be crucial for proper signal transduction of several key developmental pathways including SHH and WNT. We hypothesized that loss of *SMPD4* and disrupted cilia function would have a transcriptional effect on developing neural tissues. To test this, we performed RNA-seq of iPSCs and neural rosettes from control and *SMPD4* KO conditions and individuals with *SMPD4* deficiency. Our analysis yielded 67 differentially expressed genes in iPSC KO and 2222 genes in iPSC patient compared to iPSC control, and 74 genes in neural rosette KO and 2463 genes in neural rosette patient compared to neural rosette control (fold change≥1.5, FDR<10%; Fig. S14A-D). As the control line is isogenic to *SMPD4* KO but not *SMPD4* patient cells, we generally noted that there were not many differentially expressed genes common between KO and patient cells (Fig. S14E), potentially obscuring our further analysis. We do find this small transcriptional response surprising.

Consistent with this small number of differentially expressed genes, standard pathway analysis failed to identify common functional themes. Given the changes in cilia, we specifically examined key genes in the SHH and WNT pathways by manually selecting known key genes and/or direct targets in these pathways from the RNA-seq dataset. SHH signaling as measured by levels of *GLI1*, *SMO* and *SHH* itself is largely unaffected (Fig. S14F-H). We did note that several WNT target genes were highlighted by differential expression analysis and that WNT signaling was generally upregulated (Fig. S14I-M). We performed a pilot experiment to test whether WNT signaling was affecting cilia dynamics in the *SMPD4* iPSC models. Treatment for 48 h with the WNT antagonist IWP2 (2 μM) did not rescue ciliary length in the KO or patient cells (Fig. S15). WNT signaling is crucial for many distinct stages of neural development, suggesting an intriguing possible molecular consequence for loss of *SMPD4*.

## DISCUSSION

We have demonstrated that *SMPD4* is essential for early neural development in stem cell-derived models. We present a mouse model of loss of *Smpd4* that recapitulates human cerebellar hypoplasia but not microcephaly. Human iPSC models display cellular phenotypes consistent with disruption of forebrain development, including a novel ciliary dysfunction. Shortened primary cilia in *SMPD4* models are rescued by addition of exogenous ceramide and show evidence for dysregulated WNT signaling. Together, these results suggest that *SMPD4*-mediated sphingolipid metabolism regulates brain and primary cilia development.

### Human and mouse model discrepancies

We did not find a molecular mechanism leading to the reduced forebrain observed in the *SMPD4* germline null mouse model, although these mice rarely survive postnatally. Additionally, forebrain-specific deletion of *SMPD4* did not lead to microcephaly. Finally, a zebrafish *smpd4* loss-of-function model showed no developmental or neural abnormalities (Smits et al., 2023). However, we found strong evidence for disrupted forebrain development with our *in vitro* human iPSC models*.* Overall, these results strongly suggest a species-specific difference in the role of *SMPD4* in forebrain development. The human cortex is markedly expanded relative to other species. Developing human brains undergo several additional rounds of neural progenitor proliferation chiefly driven by radial glia-like progenitors in the outer SVZ (Hansen et al., 2010), a cortical region found in primates but not mouse. Further, the mouse brain is naturally lissencephalic (smooth), whereas the human cortex undergoes cortical folding in order to accommodate the increased number of neurons in gyrencephalic species (Subramanian et al., 2019). If the outer SVZ neural progenitors are particularly perturbed by loss of *SMPD4*, we would expect to see a more severe cortical phenotype in human than in mouse.

The cerebellar studies in mouse are a strong complement to the human *in vitro* studies presented here. The major components of cerebellar development including granule cell proliferation and Purkinje cell dendritic arbor expansion happen primarily in the early postnatal period in both mouse and humans. Human stem cell models are not yet robust enough to replace mouse models of cerebellar development. Moreover, the cerebellum is one of the most ancestral brain regions seen in both these species, which share many key developmental pathways. Thus, the role of *Smpd4* may be shared in the mouse and human but diverge in the forebrain. This apparently distinct use of *Smpd4* in the two different species remains an interesting topic for future study.

### Purkinje cells in cerebellar development

Purkinje cells are the only output cell of the cerebellum and form a circuit with the cerebellar cortex via their axonal extensions to the deep nuclei. In mouse, they are produced from E10.5-E13.5 and migrate until E17.5, when they form a monolayer underlying the EGL (Altman and Bayer, 1985a,b). The interaction between Purkinje cells and GCPs is essential for granule cell proliferation and foliation (Corrales et al., 2004; Wechsler-Reya and Scott, 1999). Postnatally, Purkinje cell survival and dendritic development significantly contribute to the cerebellar cytoarchitecture. Purkinje cells are particularly susceptible to cell degeneration and death and their loss leads to several neurodegenerative disorders including autosomal dominant cerebellar ataxias (Huang and Verbeek, 2019). Loss of *Acer3*, an alkaline ceramidase that breaks down ceramide and sphingosine-1-phosphate, causes Purkinje cell degeneration in adult mice via accumulation of long-chain ceramides and complex sphingolipids (Wang et al., 2015). Mice with a deletion in ceramide synthase (*Cers1*) have smaller Purkinje cells with underdeveloped dendritic arbors and progressively lose Purkinje cells starting at P21 (Zhao et al., 2011). The *Smpd4* null mice exhibit cerebellar hypoplasia consistent with postnatal loss of Purkinje cells. Purkinje cells in the null mouse are born in normal numbers and are present at the beginning of postnatal proliferation of GCPs but fail to survive, with apoptosis seen between P14 and P21. Because GCPs are most highly proliferative from P3-P15, this suggests that loss of the Purkinje cells (and their dendritic arbors) results in reduced trophic support for GCPs during this period and a smaller cerebellum. Why loss of *SMPD4* causes Purkinje cells to fail is a fascinating question for future work.

### Smpd3/4 have the same biochemical function but are not redundant

We hypothesized that the lack of microcephaly in the *Smpd4* forebrain conditional KO mice could be due to *Smpd3* compensatory sphingomyelinase activity in the mouse forebrain. However, this was not supported by *Smpd3*/*Smpd4* dKO experiments, nor did *Smpd3* and *Smpd4* expression in the mouse forebrain completely overlap.

No human variants in *SMPD3* have yet been published. No homozygotes with predicted loss-of-function variants, and very few homozygotes for any missense variants, are present in the Genome Aggregation Database (gnomAD), suggesting that *SMPD3* variants are not tolerated well in humans. Reported phenotypes for *Smpd3* mouse models differ. The *Smpd3^fro^* fragilitas ossium mouse displays postnatal lethality on a C57BL/6 background, runting, shorter long bones due to endochondral ossification abnormalities and decreased mineralization of cortical bones, and dentinogenesis imperfecta (Khavandgar et al., 2011). An independent *Smpd3* deletion mouse shows low embryonic lethality with postnatal growth retardation, short stature, deformed long bones with delayed ossification, and Alzheimer's-like neurological phenotypes in adulthood (Stoffel et al., 2005, 2018). The *Smpd3* deletion mouse allele reported here exhibited complete perinatal lethality with shortened and malformed long bones but no evident cortical brain abnormalities. Interestingly, previous work noted that, unlike SMPD4, SMPD3 does not associate with nucleoporins (Piët et al., 2022). This is confirmed by our subcellular localization data which shows that SMPD4 is present on the nuclear membrane, but SMPD3 is not. Therefore, the forebrain development difference between the human and mouse models is not explained by redundant neutral sphingomyelinase activity in the mouse. Collectively, these studies suggest unique subcellular roles for *SMPD3* and *SMPD4.*

### The role of SMPD4 and ceramide in centrosome dynamics, primary cilia and the cell cycle

During mitosis, the centrosomes separate by moving along the nuclear envelope to orient the mitotic spindle (Tanenbaum and Medema, 2010). Both the nuclear envelope and nuclear pore complexes break down and reassemble dynamically during mitosis (Antonin et al., 2008), which is mediated by nucleoporins (Hawryluk-Gara et al., 2008; Kutay et al., 2021). We and others have documented the subcellular localization of SMPD4 to the ER, nuclear envelope and the pericentriolar region during mitosis (Smits et al., 2023). Therefore, we hypothesize that the localization of SMPD4 and/or ceramide to the nuclear envelope is essential for interacting with the centrosome. Cell cycle disturbances have previously been shown in fibroblasts from individuals with *SMPD4* deficiency as well as HEK293T and HeLa si*SMPD4* cells (Atilla-Gokcumen et al., 2014; Magini et al., 2019; Smits et al., 2023). SMPD4 associates with nucleoporins including NUP35 and NDC1 (Piët et al., 2022). Delays in nuclear pore complex insertion and envelope closure were seen with SMPD4 knockdown (Smits et al., 2023). Nuclear pore formation defects and variants in nuclear pore proteins are known to cause microcephaly (Carapito et al., 2019; Fujita et al., 2018; Ravindran et al., 2021, 2023; Rosti et al., 2017; Sandestig et al., 2020). Nucleoporin proteins localize to the base of the primary cilium in addition to the nuclear envelope (Kee et al., 2012). This all suggests that SMPD4 localization to the nuclear membrane and centrosome contributes to its molecular role.

The centrosome also serves as the basal body for the primary cilium. The cilium is primarily known as a sensory organelle crucial for the cell to respond to intercellular signaling, but it also affects cell cycle progression (Perry and Hannun, 1998). Additionally, because ceramide is required at the basal body for primary ciliogenesis and the cilium is resorbed during S phase, primary cilia shortening is caused by cell cycle defects (Seeley and Nachury, 2010). We build upon previous work on the role of SMPD4 in controlling ceramide distribution to the nuclear envelope and primary cilium and generate further data to support a model wherein it is important for ciliogenesis. Future work will determine the molecular mechanism by which SMPD4 impacts the cell and ciliogenesis cycles. A tantalizing possibility is that an effect on WNT signaling leads to corticogenesis deficits.

We also show evidence for disrupted primary ciliary morphology in this study. We note changes in length in multiple settings upon loss of SMPD4. We also observed that the cilia in organoids sometimes have an anomalous bulbous tip. This has previously been associated with accumulation of cargo at the distal tip of the cilia secondary to disrupted retrograde ciliary transport (Tran et al., 2008). An alternative explanation may be a shedding of ciliary vesicles and ectocytosis, which potentially affects ciliary signaling. This is a process more recently appreciated in ciliary biology (e.g. Nager et al., 2017; Ojeda Naharros and Nachury, 2022; Razzauti and Laurent, 2021; Wang et al., 2021) and may be a fruitful topic for further exploration in sphingomyelinase biology. We espouse a model where the cell intrinsic effects of SMPD4 deficiency lead to primary cilia defects which then, in turn, at least contribute to the changes in the affected tissues. A different model could suppose that SMPD4 deficiency alters the tissue and the effects on cilia are in response to this primary insult. While this is a perfectly valid model and might help explain the species-specific effects on cilia we observe across different models, we prefer the first model at this time given the large body of evidence that primary cilia drive growth and differentiation of the developing nervous system.

### Local ceramide distribution may be key to SMPD4 activity

Ceramide is the precursor of many complex sphingolipids (Sandhoff and Kolter, 2003). Ceramide is synthesized primarily via the ‘hydrolysis’ pathway (Gillard et al., 1998) in the cell membrane, *de novo* in the smooth ER and, rarely, through the ‘salvage’ pathway in the lysosome. Although *SMPD4* knockdown or loss-of-function does not lead to accumulation of sphingomyelin or depletion of ceramide at the tissue level (Atilla-Gokcumen et al., 2014; Smits et al., 2023; this study), organelle localization of these sphingolipids is key to their activities (Airola and Hannun, 2013). Localized lipid distribution in organelle membranes, such as ceramide, changes the membrane curvature (Arya et al., 2022). This study also suggests that neutral sphingomyelinase activity is needed for nuclear envelope buds to release and form cytosolic vesicles. Because ceramide biogenesis is thus specific to certain organelle membranes, we hypothesize that nuclear membrane loss of SMPD4 could lead to a local deficit in ceramide, perturbed local concentrations and compromised transport to the plasma membrane. This would also result in a deficit of ceramide available at the plasma membrane, where acetylated tubulin associates with ceramide-rich platforms for primary ciliogenesis (Tripathi et al., 2021). Bulk ceramide supplementation is a candidate intervention, but there is no established paradigm for this. While this seems to be a promising avenue for future investigation, issues such as distribution through a three-dimensional system and the blood–brain barrier may prove problematic. We demonstrate that ceramide supplementation can rescue cilia length, but emphasize that our paradigm was a two-dimensional cell culture system. This may be more akin to a significant overexpression leading to localized concentration changes, which may not be measurable with current methods but are the actual key signaling events.

### Limitations to the study

There are some features of the experimental design and data reported here that limit some of the conclusions that can be drawn. While the human stem cell data are intriguing and give some insight into the *SMPD4* disease etiology, some further additions would have been preferable. For reasons we cannot fully explain, we were unable to generate an isogenic cell line wherein the patient variant was ‘rescued’ back to WT with the use of genome editing tools. In addition to this being an invaluable control when possible to obtain, the different ethnicity of the patient as compared to the donor of the other cell lines resulted in a large number of genes differentially expressed in the RNA-seq analysis that were likely not due to any biological functions of *SMPD4.* We also worked with one deletion cell line (*SMPD4 KO*). As standards for the stem cell community have evolved, we now appreciate that multiple clones for each genotype would be preferred. These guidelines and others are evolving from the community and standards were published by the International Society for Stem Cell Research in 2023, near the completion of the work reported here. Finally, we note a striking difference in the effects of *SMPD4* loss between the human and mouse models. While the data about cilia length are different between models, our findings are consistent within the data for each model system. This suggests that further work is needed to fully understand the role of *Smpd4* development and ciliary biology.

### SMPD4 as a disease gene

In this work, we show a new role for *SMPD4* in the primary cilium. We link primary cilia length to ceramide, expanding our understanding of how deficits in neutral sphingomyelinase cause brain disorders. We have advanced the study of *SMPD4* as an interesting cause of a human structural brain disorder. This study suggests that modulating *SMPD4*, neutral sphingomyelinase function, and/or ceramide content in the cell would improve brain development in human patients.

## MATERIALS AND METHODS

### Generation of KO mouse alleles

Mouse zygotes (C57BL6/N strain) were injected with 200 ng/µl CAS9 protein (Thermo Fisher Scientific) and 100 ng/µl *Smpd3*-specific sgRNA (TGGCCAGAGCAGGCTGCACGCGG, CAGGTCCTAAAGCAGCAGTCAGG, Integrated DNA Technologies), followed by surgical implantation into pseudo-pregnant female (CD-1 strain) mice [Cincinnati Children's Hospital Medical Center (CCHMC) Transgenic Animal and Genome Editing Core (TAGE), RRID:SCR_022642]. The resulting live-born pups were weaned and used for additional PCR screening and mating.

*Smpd4* mice were obtained from the International Mouse Phenotyping Consortium (IMPC) (RRID:SCR_006158, *Smpd4*^tm2a(KOMP)Wtsi^). The *tm2a* allele can be converted to *tm2b* (null) or *tm2c* (flox) in this system. We generated *tm2b* (null) mice by crossing *tm2a* with a germline Cre (EIIa-Cre, The Jackson Laboratory, RRID:SCR_004633), and *tm2c* (flox) mice by crossing with FLP carrier mice (The Jackson Laboratory). Conditional Cre alleles were from The Jackson Laboratory. *Smpd4^null/+^*; *Cre^+^* mice were crossed with *Smpd4^flox/flox^* to generate *Cre^+/WT^; Smpd4^flox/null^* mutants, which we refer to as conditional KO mice. A list of mouse alleles used for this project is in Table S1.

### Mouse husbandry

All animals were maintained through a protocol approved by Nationwide Children's Hospital Medical Center Institutional Animal Care and Use Committee (IACUC2021-AR1200067). Mice were housed with a 12 h light/12 h dark cycle with food and water *ad libitum*. Mouse euthanasia was performed in a carbon dioxide chamber followed by secondary cervical dislocation. Genotyping was performed via PCR and gel electrophoresis on a 2% agarose gel with the primers listed in Table S2. Whole-brain and skeletal images were taken using a Zeiss Discovery V12 microscope.

### Behavior testing

Hindlimb clasping was measured on P21 by recording a 10 s video on a camcorder while the animal was suspended by the tail ∼15 cm from the cage floor. Two researchers unaware of genotype independently scored tail clasping on a 0-4 scale (Lukacs et al., 2019) and the average of the two measurements was used for statistical analysis. Motor coordination was tested at 6 weeks of age (*n*=9-10 animals per genotype) on a Rotor-Rod™ (San Diego Instruments) with speed increasing from 0 to 50 rpm over a 2 min testing period. The researcher was again unaware of genotype. The Rotor-Rod™ system measured distance traveled (cm) and latency to fall (s). An even number of males and females were tested per genotype for both behavior tests, and we note there was no statistical difference between sexes. Three trials were performed per animal with a 15 min break between each trial.

### Histology

Brains were dissected, fixed in formalin for 48 h, washed in 70% ethanol, then dehydrated and paraffin-embedded by the CCHMC Integrated Pathology Research Facility. Blocks were sectioned on a microtome at 10 µm (Sakura). Sections were placed on glass slides (Cardinal Health), baked for more than 1 h, and stained with Hematoxylin and Eosin using standard methods. Body and brain weights were obtained on a standard chemical scale. For cortical and cerebellar measurements, area in µm^2^ or length in μm was measured on ZEN 3.7 software. A minimum of three animals from at least two distinct litters were measured for each genotype.

### Skeletal preparation

Pups were collected at E18.5 and frozen. Skin and fat were removed from the embryos before fixation in 95% ethanol for 2-5 days. The skeletons were stained with Alizarin Red and Alcian Blue and cleared with potassium hydroxide using standard methods (Behringer et al., 2014). Bone length measurements were taken using ZEN 3.7 software (*n*=3-4 animals per genotype).

### Immortalized cell culture

HEK293T cells (ATCC) were cultured in Dulbecco's Modified Eagle Medium (DMEM) supplemented with 10% fetal bovine serum and 1% penicillin/streptomycin at 37°C and 5% CO_2_. Cells were enzymatically detached from plates using 1 ml trypsin/EDTA (0.25%). Plasmid transfections used the Lipofectamine 3000 kit following the manufacturer's instructions (Thermo Fisher Scientific). A control GFP plasmid was used to assess transfection efficiency at 48 h post-transfection (GFP-Nubp1, Okuno et al., 2010).

### iPSC culture

Fibroblasts were obtained from an affected individual with *SMPD4* variant p.Glu124* (Magini et al., 2019) (Family 8) and reprogrammed to pluripotency by the CCHMC Pluripotent Stem Cell Facility (PSCF) (RRID: SCR_022634) using standard protocols (McCracken et al., 2014). Control human iPSCs (Episomal hiPSC, Gibco) and patient-derived iPSCs were cultured in mTeSR media (StemCell Technologies) in Nunc plates (Fisher) on a matrix of Matrigel (Corning) dissolved in DMEM/F12. Cells were passaged every 7 days using Gentle Cell Dissociation Reagent (StemCell Technologies) and fed daily. For exogenous treatment experiments, iPSCs were plated onto Matrigel-coated coverslips in a 24-well plate. Cells were treated with Fumonisin B1 (Sigma-Aldrich) at 30 µM for 72 h, GW4869 (Tocris Bioscience) at 10 µM for 72 h, C16 ceramide (Avanti Polar Lipids) at 2 µM for 48 h, ACY1215 (APExBIO) at 1 µM for 18 h and/or IWP2 (Tocris Bioscience) at 2 µM for 48 h before being processed for immunocytochemistry.

The STEMdiff™ SMADi Neural Induction Kit (StemCell Technologies) was used to generate neural rosettes from high-quality iPSC colonies. Briefly, iPSCs were dissociated into single cells and plated into an Aggrewell 800 well (StemCell Technologies) at 10,000 cells per well, forming embryoid bodies. These were fed daily and replated on day 5 by filtering through a 37 µM reversible strainer (StemCell Technologies) into a 24-well plate containing coverslips coated in Matrigel. On day 8, percent neural rosette formation was visually confirmed to be 75% or above before harvesting for immunocytochemistry.

Whole-brain neural organoids were generated from an adapted protocol based on Fair et al. (2023). Cells were dissociated as above and resuspended in mTesR in 96-well ultra-low attachment plates on day 0. On day 10, embryoid bodies were transferred into six-well plates and maintained until day 28, when organoids were fixed overnight in 4% paraformaldehyde (PFA) at 4°C and prepared for cryo-embedding and immunohistochemistry as previously described (Fair et al., 2023).

### Directed trilineage differentiation assay

To assess functional pluripotency, iPSCs were subjected to directed *in-vitro* trilineage differentiation to ectoderm, definitive endoderm and mesoderm, and co-expression of lineage markers was assessed by immunofluorescence staining at the conclusion of experiments. Cells were dissociated to single cells using Accutase (StemCell Technologies, 07920) and seeded in mTeSR1 containing 10 µM Y27632 at 4E+05 (ectoderm), 1E+05 (mesoderm) and 1.5E+05 (endoderm) cells per well in 24-well cell culture plates. Each well contained a Cultrex-SCQ (Bio-techne, 3434-010-02) coated coverslip (Thermo Fisher Scientific, 174950). Directed differentiation was initiated 16 h after seeding cells. Ectoderm and mesoderm differentiation used STEMdiff Trilineage Ectoderm Medium (StemCell Technologies, 05231) and STEMdiff Trilineage Mesoderm Medium (StemCell Technologies, 05232), respectively, according to the manufacturer's instructions. Definitive endoderm differentiation was performed as previously described (Pitstick et al., 2022). Plates containing undifferentiated controls and cells subjected to differentiation were washed with Dulbecco's Phosphate-Buffered Saline (DPBS) and fixed for 15 min at room temperature using 4% PFA in DPBS. Plates containing fixed cells were wrapped in parafilm and stored at 4°C for a maximum of 7 days before immunostaining.

For immunostaining, cells were incubated in permeabilization buffer (0.5% Triton X-100 in DPBS) for 15 min at room temperature. Cells were then incubated for 30 min at room temperature in a blocking solution containing 5% normal goat serum (NGS) in permeabilization buffer and then overnight at 4°C with primary antibodies directed against the stemness marker OCT4 (Santa Cruz Biotechnology, sc5279, 1:500), ectoderm markers PAX6 (BioLegend, 901301, 1:500) and SOX2 (R&D Systems, AF2018, 1:500), endoderm markers SOX17 (R&D Systems, AF1924, 1:500) and FOXA2 (Abcam, ab108422, 1:500), and mesoderm markers CDX2 (Cell Marque, 235R-14, 1:500) and Brachyury (R&D Systems, AF2085, 1:500). Primary antibodies were diluted in blocking buffer. The following day, cells were washed with DPBS and incubated for 45 min with fluorophore-labeled secondary antibodies diluted 1:500 in blocking buffer (Thermo Fisher Scientific, A-31571, A10040 and A111055). After washing in DPBS and counterstaining DNA with 4′,6-diamidino-2-phenylindole (DAPI), coverslips were mounted on glass slides in Fluoromount-G Mounting Medium (Southern Biotech, 0100-01) and imaged by confocal microscopy.

### Mycoplasma detection

Cell cultures were tested for mycoplasma contamination with the MycoAlert Mycoplasma Detection Kit (Lonza) according to the manufacturer's instructions.

### CRISPR/Cas9 transfection

CRISPR guides were purchased from Synthego. CRISPR editing was performed on control iPSCs using the Synthego CRISPR Lipofection protocol with GFP control as described above. Cells were serially diluted into a 96-well plate (10,000 cells/plate) for clonal isolation. Wells with only a single colony were grown to confluency, passaged into six-well plates, and DNA was extracted for PCR to confirm CRISPR editing of the *SMPD4* locus. PCR product was purified using the DNA Clean & Concentrator kit (Zymo Research) and Sanger sequenced (Fig. S6). CRISPR guides and primers for genotyping are in Table S2.

### Immunocytochemistry

Cells were plated onto coverslips in a 24-well tissue culture plate. They were fixed in 4% PFA for 15 min. Coverslips were immersed in 0.1% Triton X-100 for 5 min before blocking to permeabilize cell membranes. Coverslips were blocked in 4% NGS for 30 min and incubated at 4°C overnight in primary antibodies. Secondary antibody staining for 1 h was followed by DAPI incubation for 15 min. Coverslips were sealed to glass slides with ProLong Gold Antifade Mountant (Thermo Fisher Scientific). Images were acquired using a Zeiss Axio Imager.M2 with Apotome 3 and an Axiocam 305 monochrome camera. Antibodies and concentrations for immunocytochemistry are listed in Table S3. CC3^+^, PHH3^+^ and PAX6^+^ cells from embryonic mice, neural rosettes and organoids were counted in the NIS-Elements Analysis program (Nikon) with three images quantified for each of *n*=3 replicates. Calbindin^+^ cells were quantified by the same method but with one image from each animal (*n*=3-9 per genotype).

For analysis of primary cilia, cells were serum-starved in Opti-MEM Reduced-Serum Medium (Thermo Fisher Scientific) for 24 h before fixation in methanol. *Z*-stack images were taken on the same Zeiss Apotome at 63× magnification and then a maximum intensity projection was produced from the *z*-stack. Cilia were quantified using an NIS-Elements Analysis program, with ∼1000 cells per replicate per condition. Cilia length was measured in µm with ZEN 3.7 using the maximum intensity projection images, 20-30 cilia per image with *n*=3 replicates per genotype. The transfection experiment and cell counting were repeated (*n*=3) for each condition.

### Immunohistochemistry

After dissection, brains were fixed for 1-2 days in PFA at 4°C. PFA was replaced with 30% sucrose for 2 days before brains were embedded in Optimal Cutting Temperature solution (Sakura) and stored at −80°C. Then 10 µm sections were obtained for mouse brain and human organoid samples using a Leica CM 1860 cryostat, placed on glass slides, and stored at −20°C. Slides selected for immunohistochemistry were pre-warmed at 42°C for 10-15 min. Antibody retrieval was performed as previously described (Bittermann et al., 2019). Blocking and secondary antibody processing was as described above.

### RNA *in situ* hybridization

WT mouse embryos maintained on a CD1 genetic background were dissected at ages E12.5, E14.5, E18.5, P0 and P28 and fixed in formalin for 16-24 h; brains were sub-dissected at ages E18.5-P27. Tissue was washed in PBS, then dehydrated and paraffin-embedded by the morphology core. Paraffin blocks were sectioned at 5 µm, placed on glass slides and baked at 60°C for 1 h. Target retrieval steps outlined in the ACDBio RNAScope protocol were followed based on recommendations for brain tissue, then slides were dried at room temperature overnight. Hybridization and amplification steps were performed using the HybEZ oven set at 40°C, RNAscope Multiplex Fluorescent Reagent Kit V2 (323100), TSA Cyanine 3 Fluorophores (NEL744001KT) at 1:750, and *Smpd4* probe custom designed by ACDBio.

### Mass spectrometry

Liquid chromatography-electrospray ionization/mass spectrometry (LC-ESI-MS/MS) was performed (Ho et al., 2003). Crude lipid extracts were prepared from mouse brain tissue using a single-phase extraction system of ethyl acetate:isopropanol:water. Along with internal standards, the extract was passed through an HPLC system coupled to a mass spectrometer with electrospray ionization capability (ESI/MS) with precursor ion scan and multiple reaction monitoring enabled. Each decomposed sphingolipid was then quantified by comparing to internal standards.

### RNA/WGS sequencing

Total RNA was extracted from iPSCs or neural rosettes using the Trizol (Tri Reagent, Sigma-Aldrich) protocol with post-extraction cleanup. Samples were kept on ice until storage at −80°C. RNA concentration was quantified via Nanodrop (Thermo Fisher Scientific). DNA was extracted from tail clips with a DNeasy Blood & Tissue Kit (Qiagen) for whole genome sequencing (WGS). DNA concentration was quantified via Qubit. RNA-seq and WGS were performed by the Nationwide Children's Hospital Genomics Services Laboratory using standard Illumina protocols. RNA-seq was performed in triplicate for all three conditions, including the control cell lines.

### Statistical analysis

Data plots and subsequent analyses were performed with Prism 9 (GraphPad). A Chi-square test was used for genotypic ratios of mice studied. An unpaired two-tailed Student's *t*-test was performed for experiments with two groups. A one-way ANOVA with Tukey's multiple comparison tests was performed for experiments with more than two comparisons. ANOVA *P*-values are usually stated in the text or figure legend and specific relevant *P*-values for the multiple comparisons are shown in the relevant figure. We report the statistical test values directly rather than assigning a significance symbol to provide all the data for the reader. Data shown are the median or median±95% confidence interval.

## Supplementary Material



10.1242/develop.202645_sup1 Supplementary information
