# Supplementary material for: SMPD4-mediated sphingolipid metabolism regulates brain and primary cilia development
Source: Development. 2024 Nov 15;151(22):dev202645. doi: 10.1242/dev.202645 (PMC11586524; doi:10.1242/dev.202645)
Supplement: Supplementary information [file develop-151-202645-s1.pdf]

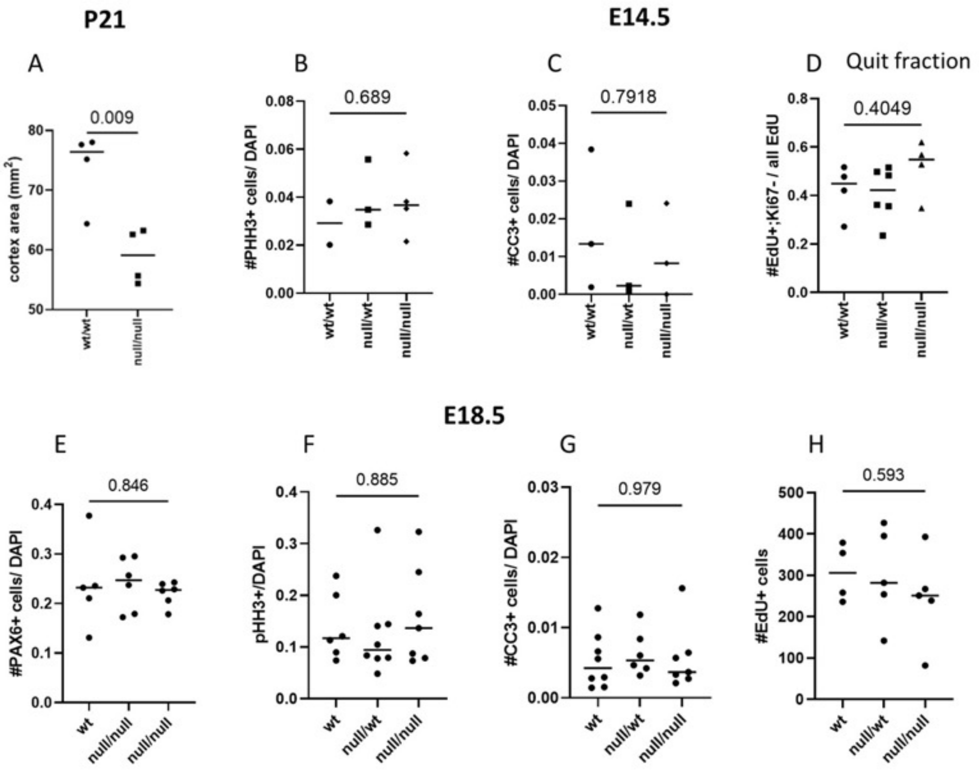

Fig. S1. Normal proliferation and cell death in the *Smpd4* homozygous null forebrain. The cortical area of weaning stage *Smpd4*$^{null/null}$ animals was found to be smaller than their littermates (n=4 wild-type, 4 control, p =0.009; A) in addition to having a smaller body weight and failure to thrive. Next, forebrains from E14.5 *Smpd4* wild-type, heterozygous, and homozygous null animals were analyzed for several molecular markers. Quantification of pHH3-positive cells indicates no difference in proliferation (n=2 wild-type, 3 null/wt, 4 null/null, ANOVA p =0.689; B) and the number of CC3-positive apoptotic cells in the ventricular zone show no elevated cell death (n=3 wild-type, 3 null/wt, 2 null/null, ANOVA p =0.792; C). Dividing cells at E13.5 were labeled via i.p. injection of EdU into the pregnant dam and embryos were analyzed at E14.5. Sections were stained with Ki67 to mark cells actively dividing at E14.5 24 hours after the EdU label. Cells that are EdU-positive and negative for Ki67 have left the cell cycle (the so-called quit fraction). An elevated number of such cells could help explain a smaller cortex. We did not detect a markedly different number of Edu-positive, Ki67-negative cells within the EdU-positive population in mutants compared to wild-type (n=4 wild-type, 6 null/wt, 4 null/null, ANOVA p =0.405; D). Similarly at E18.5, the number of PAX6-positive neural progenitors in the cortex (n=5 wild-type, 6 null/wt, 6 null/null, ANOVA p =0.846; E) is unaffected, as are the number of pHH3-positive (n=6 wild-type, 8 null/wt, 7 null/null, ANOVA p =0.885; F), CC3-positive (n=8 wild-type, 6 null/wt, 7 null/null, ANOVA p =0.979; G), and E13.5 labeled EdU-positive cells (n=4 wild-type, 5 null/wt, 5 null/null, ANOVA p =0.593; H).

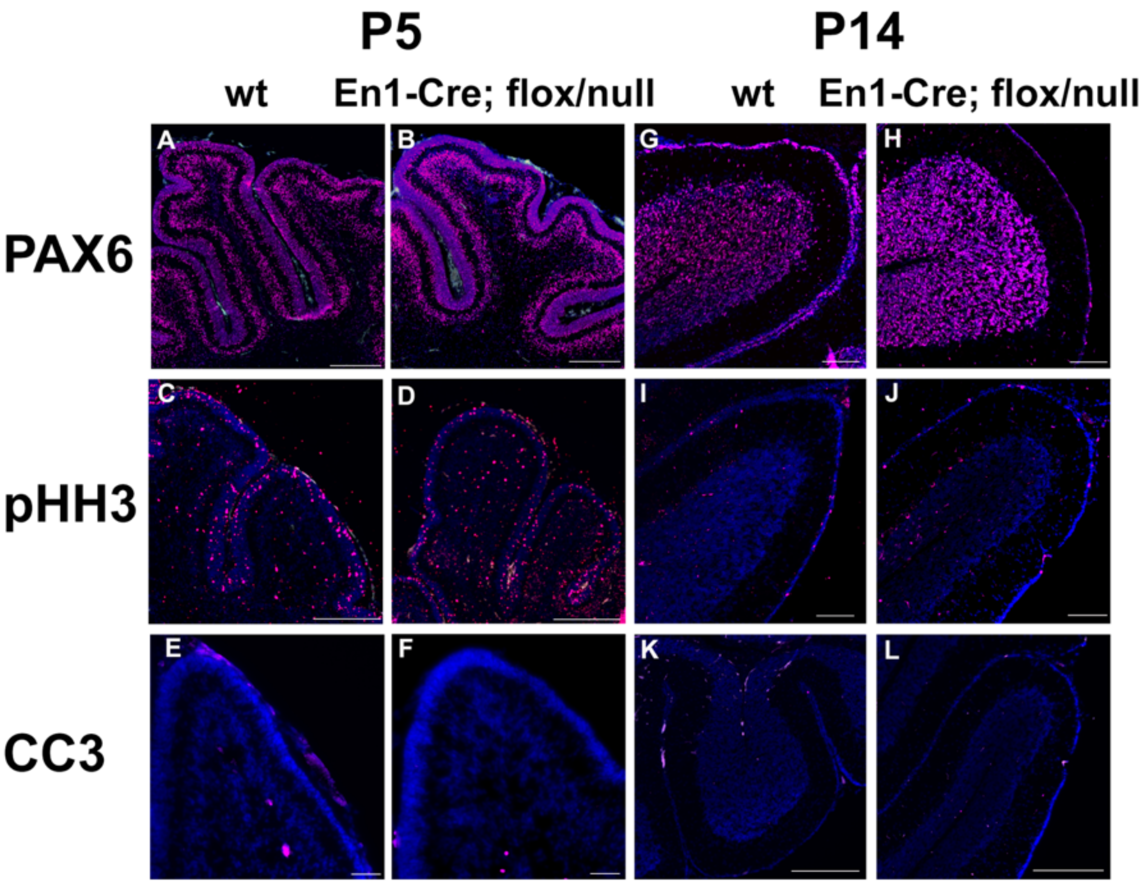

Fig. S2. Neurogenesis markers in En1-Cre+; *Smpd4*$^{flox/null}$ animals. Immunohistochemistry at P5 (A-F) and P14 (G-L) for PAX6 (A,B,G,H), pHH3 (C,D,I,J) and CC3 (E,F,K,L). Scale bars = 200μm A-D, 50μm E,F, 100μm G-L.

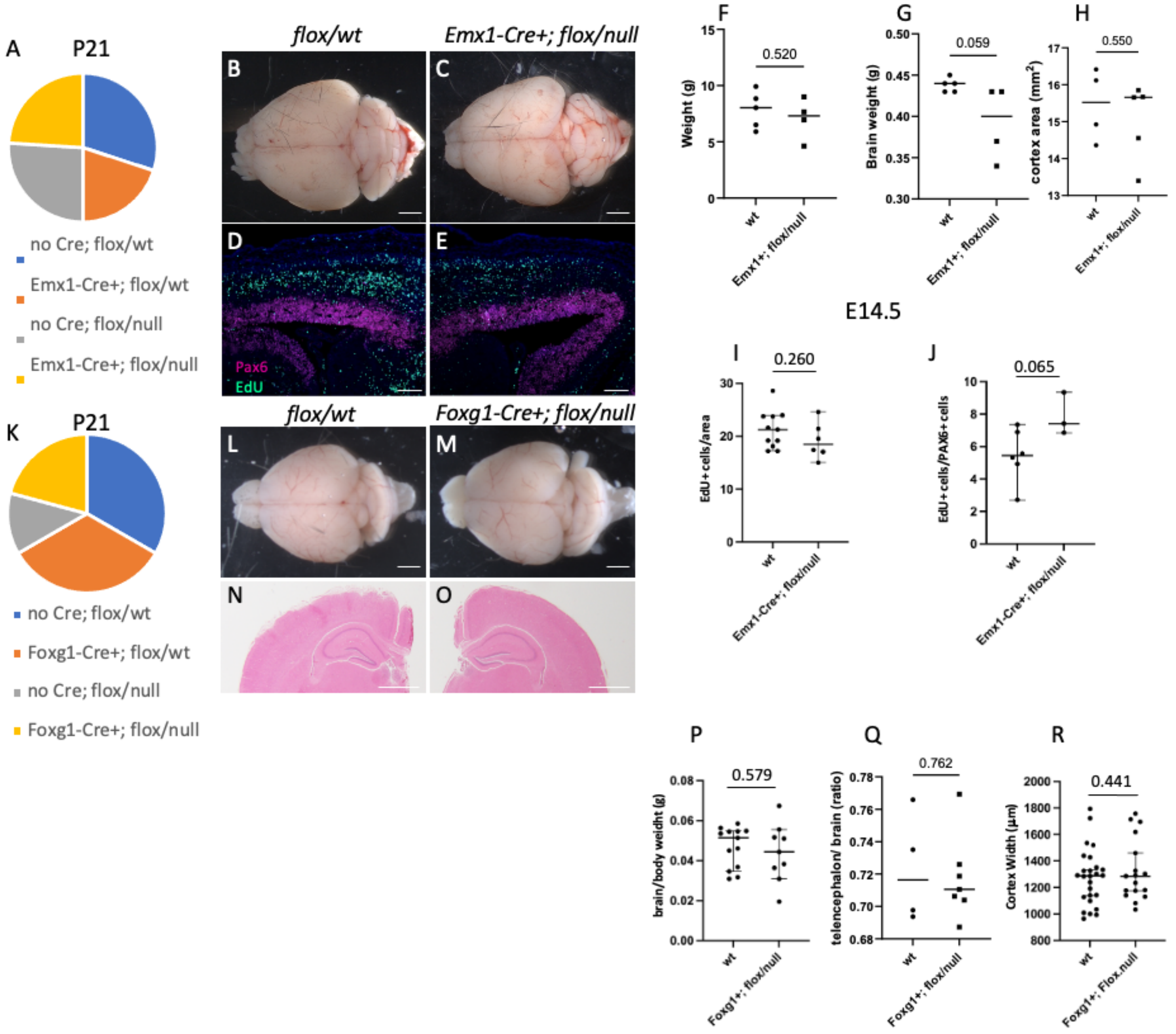

Fig. S3. Conditional forebrain ablation of *Smpd4* rescues survival but does not recapitulate microcephaly. *Emx1-Cre+; Smpd4*$^{flox/null}$ animals survive in Mendelian ratios at weaning (A) and do not exhibit any gross cortical abnormalities (B-E). They are the same size as their littermates (n=4 wild-type, 4 conditional deletion, p=0.520; F) and demonstrate a reduction in brain weight (n=5 wild-type, 4 conditional deletion, p=0.059; G), but not cortical area (n=4 wild-type, 5 conditional deletion, p=0.550; H). Injection of pregnant females with EdU at E13.5 and dissection of embryos at E14.5 suggest normal cortical migration (D-E, quantified in I-J). (I: n=11 wild-type, 6 conditional deletion, p=0.260; J: n=6 wild-type, 3 conditional deletion, p=0.065) Similar results were obtained for *Foxg1-Cre+; Smpd4*$^{flox/null}$ mice (K-R; P: n=6 wild-type, 9 conditional deletion, p=0.579; Q: n=4 wild-type, 7 conditional deletion, p=0.762; R: n=26 wild-type, 17 conditional deletion, p=0.441). Scale bars in B,C,L,M = 2mm, D,E = 100µm, N,O = 1mm.

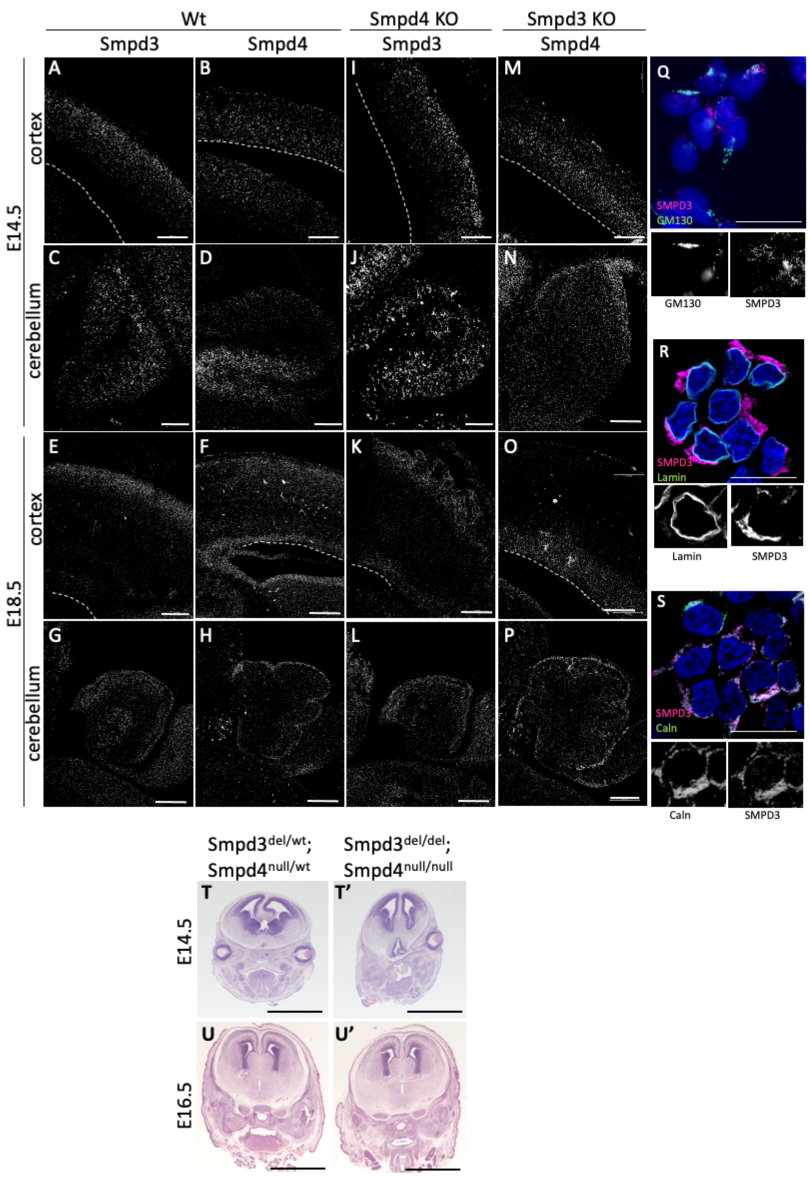

Fig. S4. Neutral sphingomyelinases do not compensate for each other in mouse. RNA *in situ* hybridization shows that at E14.5 and E18.5, *Smpd3* is restricted to upper cortical layers in the forebrain (A,B) but is expressed throughout the cerebellar primordium (C,D). Compensation of neutral sphingomyelinases was tested by assaying *Smpd3* mRNA in the absence of *Smpd4* (*Smpd4*$^{null/null}$, I-L) and *Smpd4* mRNA in *Smpd3*$^{del/del}$ cortex, finding no upregulation in mRNA expression in either case. Subcellularly, SMPD3 protein does not localize to the GM130-positive Golgi apparatus (Q) or Lamin-positive nuclear membrane (R) but is localized to the Calnexin-positive ER (S). Double knockout *Smpd3*$^{del/del}$; *Smpd4*$^{null/null}$ animals do not exhibit any obvious craniofacial abnormalities and their cortical width is unchanged at E14.5 and E16.5 (T,U). Scale bar A-P = 200µm, Q,R,S = 20 µm, T-U' = 2mm.

Development • Supplementary information

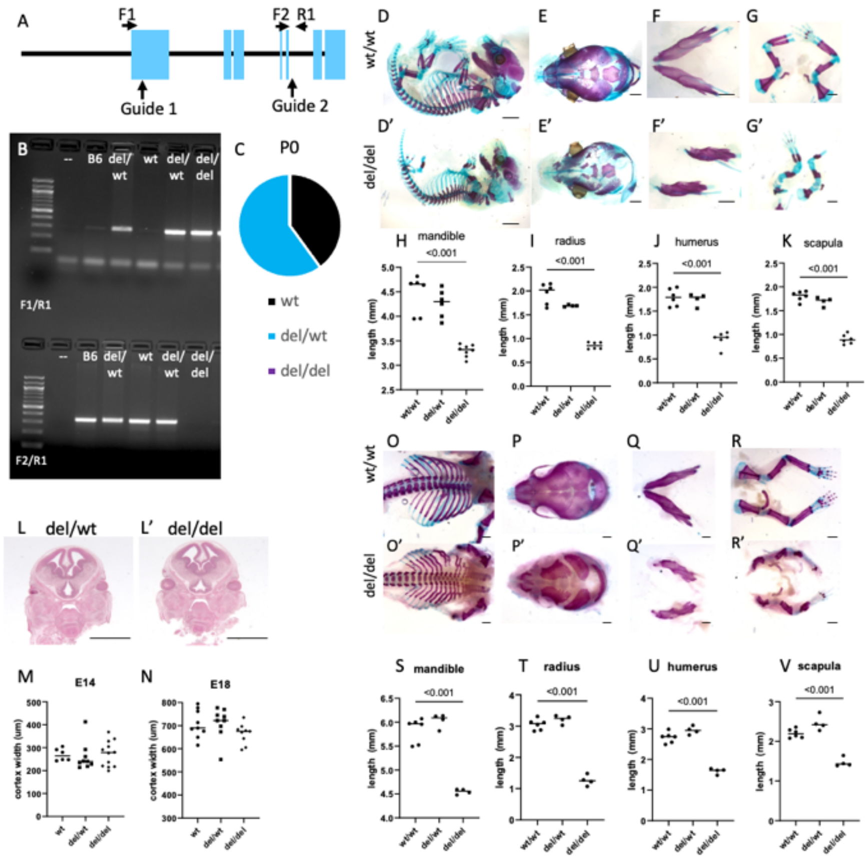

Fig. S5. Generation of a novel *Smpd3* allele reveals requirement for postnatal survival and skeletal development but not brain development. CRISPR/ Cas9 guides were designed to induce a large deletion in *Smpd3* (A). Primers to genotype resulting animals are schematized (A) and results are shown in B. A PCR product of primers F1 and R1 indicates deletion of exons 1-5. *Smpd3* homozygous deletion animals do not survive at birth (C). Skeletal preps from E18.5 animals show overall dysmorphic and shortened bones in homozygous deletion animals (D-G, mandible n=6 wild-type, 6 deletion/wt; 8 deletion/deletion, ANOVA p<0.0001, H; radius n=6 wild-type, 4 deletion/wt; 6 deletion/deletion, ANOVA p<0.0001, I; humerus n=6 wild-type, 4 deletion/wt; 6 deletion/deletion, ANOVA p<0.0001, J; scapula n=6 wild-type, 4 deletion/wt; 6 deletion/deletion, ANOVA p<0.0001, K). However, coronal sections at E14.5 (L; n=6 wild-type, 9 deletion/wt; 12 deletion/deletion, ANOVA p=0.0801, M) and E18.5 (n=9 wild-type, 9 deletion/wt; 9 deletion/deletion, ANOVA p=0.194, N) do not show brain abnormalities or cortical thinning. We confirmed the phenotype is the same on an outbred CD1 strain (O-V, mandible n=6 wild-type, 4 deletion/wt; 4 deletion/deletion, ANOVA p<0.0001, S; radius n=6 wild-type, 4 deletion/wt; 4 deletion/deletion, ANOVA p<0.0001, T; humerus n=6 wild-type, 4 deletion/wt; 4 deletion/deletion, ANOVA p<0.0001, U; scapula n=6 wild-type, 4 deletion/wt; 4 deletion/deletion, ANOVA p<0.0001, V). Scale bars in D,D',L,L' = 2mm, E,G', O-R' = 1 mm.

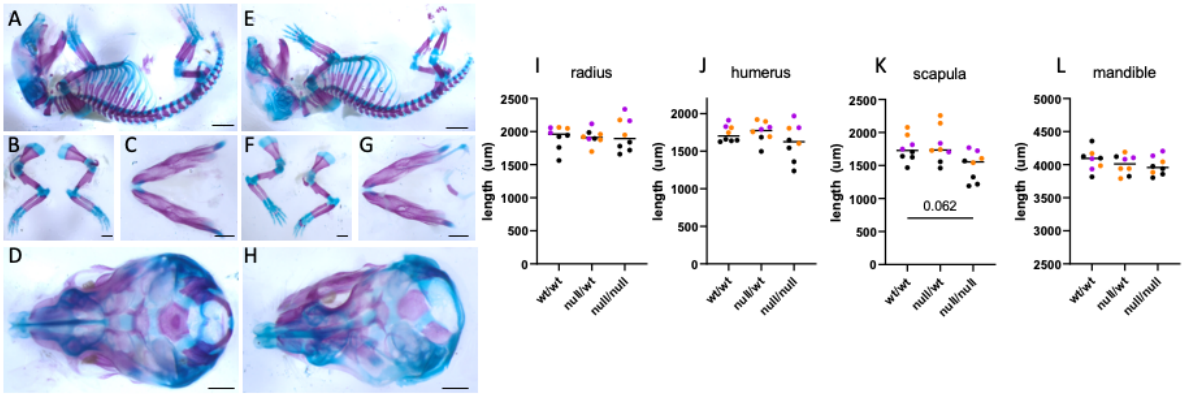

Fig. S6. *Smpd4* mice do not exhibit skeletal abnormalities. E18.5 skeletons of *Smpd4* wild-type (A-D) and homozygous null (E-H) show no differences in bone (Alizarin Red) or cartilage (Alcian blue) appearance. The lengths of various long bones and the mandible are shown (radius n=8 wild-type, 8 deletion/wt; 8 deletion/deletion, ANOVA p=0.911, I; humerus n=8 wild-type, 8 deletion/wt; 8 deletion/deletion, ANOVA p=0.268, J; scapula n=8 wild-type, 8 deletion/wt; 8 deletion/deletion, ANOVA p=0.0303, K; mandible n=8 wild-type, 8 deletion/wt; 8 deletion/deletion, ANOVA p=0.447, L). Scale bar A,E = 2mm, B-D, F-H = 1mm.

A

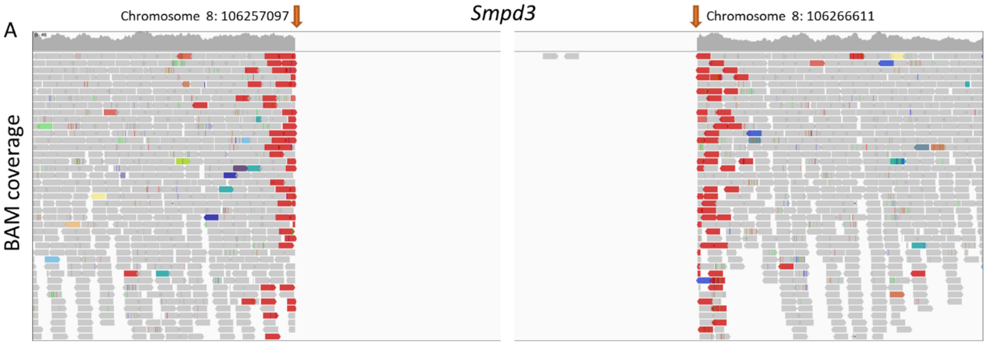

B

Chr	Start	End	Length	SV Type	Callers	Genes
1	1.52E+08	1.52E+08	8158	DEL	3	Gm28181
1	1.67E+08	1.67E+08	6389	DEL	3	Gm22992
1	1.71E+08	1.71E+08	11520	DEL	3	Gm37154,Pfdn2,Nit1
1	1.57E+08	1.57E+08	2712	DEL	3	Csnk2a3
1	1.63E+08	1.63E+08	7113	DEL	4	Fmo1
1	1.35E+08	1.35E+08	1431	DEL	3	Gm4204
1	1.34E+08	1.34E+08	5522	DEL	3	Cyb5r1
1	1.28E+08	1.28E+08	1767	DEL	4	R3hdm1
1	1.47E+08	1.47E+08	4596	DEL	3	Gm15583
1	1.49E+08	1.49E+08	2851	DEL	3	Gm15443
1	1.41E+08	1.41E+08	7215	DEL	3	Gm37371
1	1.44E+08	1.44E+08	6136	DEL	3	Glrx2
3	1.07E+08	1.07E+08	1250	DEL	3	Slc6a17
3	1.38E+08	1.38E+08	2961	DEL	3	Adh5
4	1.45E+08	1.45E+08	2767	DEL	3	Gm13127
4	78658763	78661837	3074	DEL	3	Gm11261
4	91851467	91852743	1276	DEL	3	Gm12671
4	83504841	83506760	1919	DEL	3	Gm10154
4	44139387	44141254	1867	DEL	3	Rnf38
4	10486893	10489688	2795	DEL	3	Gm11815
4	54655412	54662068	6656	DEL	3	Gm12480
4	51252473	51254391	1918	DEL	3	Amd-ps4
4	55418205	55423330	5125	DEL	3	Gm12505
4	3994609	3997118	2509	DEL	3	Sdr16c5
4	25560135	25566886	6751	DEL	3	Gm24977
6	1.17E+08	1.17E+08	6422	DEL	3	Tmem72
6	1.16E+08	1.16E+08	5512	DEL	3	Gm44206
6	1.05E+08	1.05E+08	4168	DEL	4	Gm19757
6	1.29E+08	1.29E+08	9997	DEL	3	Gm27336,Clec2g
6	1.23E+08	1.23E+08	3606	DEL	4	Nanog
6	74447053	74451905	4852	DEL	3	Gm23924
6	78395406	78403705	8299	DEL	3	Reg2
7	1.04E+08	1.04E+08	4816	DEL	3	Gm25405
7	24097894	24101446	3552	DEL	3	Zfp180
8	1.06E+08	1.06E+08	9514	DEL	4	Smpd3
10	93778609	93779791	1182	DEL	3	Gm3571
10	42052544	42055477	2933	DEL	3	Tdg-ps2
10	82585380	82587550	2170	DEL	3	Ap3m1-ps
10	25622969	25629411	6442	DEL	3	Gm47924
10	25895608	25904027	8419	DEL	3	Gm47939
12	69580011	69581118	1107	DEL	3	Vcpkmt
12	1.01E+08	1.01E+08	18025	DEL	3	Rps6ka5
13	23684621	23692177	7556	DEL	3	H2bc4
15	92025993	92028124	2131	DEL	3	Gm3888
15	1.04E+08	1.04E+08	6907	DEL	3	Gm49272-5
18	80218804	80222175	3371	DEL	3	Txnl4a
18	43472653	43477860	5207	DEL	3	Gm27325,Gm3650,Eif3j2
18	37720964	37731566	10602	DEL	3	Pcdhgb4,a8,b5,Gm37165
18	48046645	48048413	1768	DEL	3	Eno1b
18	14570097	14571714	1617	DEL	3	Gm6457
19	40621778	40624608	2830	DEL	3	Gm8783
19	24875624	24876881	1257	DEL	3	Gm10053
19	21937250	21938594	1344	DEL	3	Ldhb-ps

Fig. S7. *Smpd3* homozygous mouse sequencing shows a specific large deletion. Concurrent with our Sanger sequencing confirmation of CRISPR/Cas9-mediated editing, whole genome sequencing of a *Smpd3*$^{del/del}$ mouse indicated a large 9,514 base-pair deletion corresponding to exons 3-7 of *Smpd3*. Read sequence alignment is shown in A. There were no other appreciable large deletions/ structural variants on chromosome 8 (B).

Development • Supplementary information

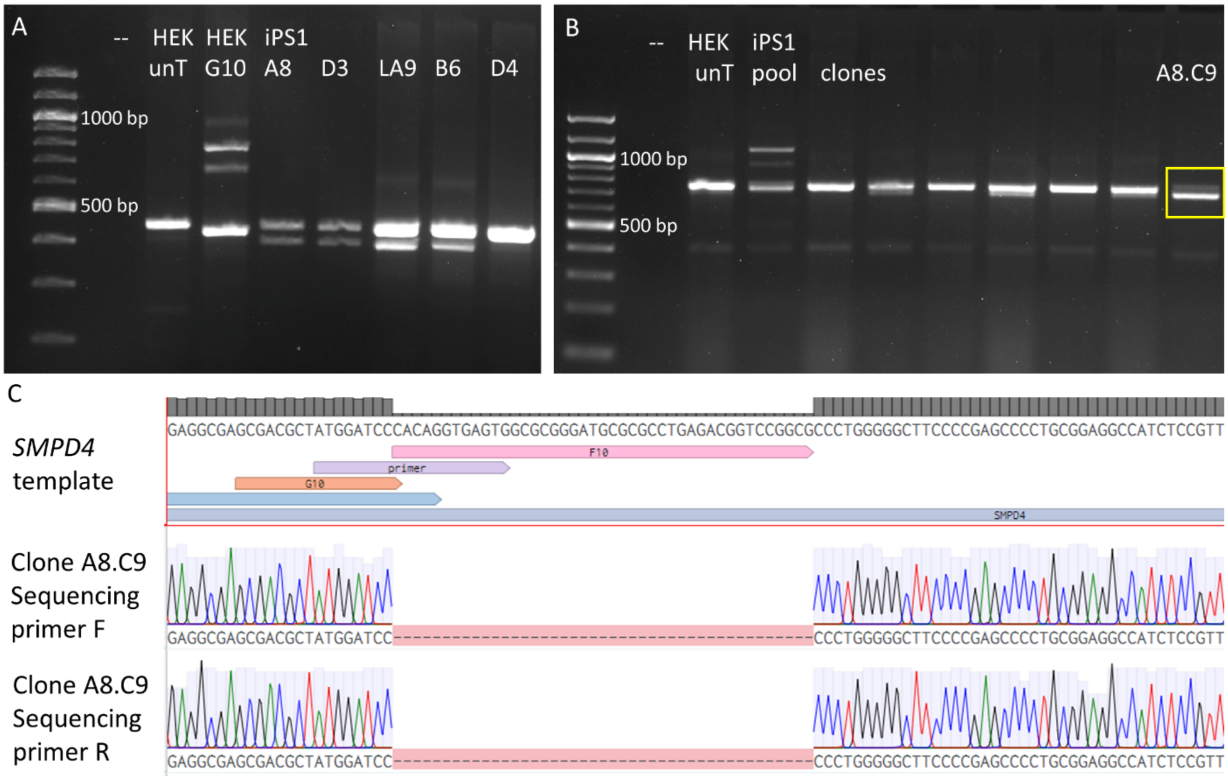

Fig. S8. CRISPR Editing of *SMPD4* Locus in human iPSCs. CRISPR/Cas9 editing was used to disrupt the end of Exon 1 of *SMPD4*. CRISPR guides in an RNP complex designed by Synthego were transfected into control iPSCs. PCR of single clones showed a promising edited lower band for several clones including A8 (A) whereas clone D4 was unedited (HEK=HEK293T cell line; iPS1= control iPS line; unt= untransfected). Sanger sequencing for clone A8 showed a heterozygous 43 base pair deletion in *SMPD4*. The CRISPR experiment was repeated on this clone to edit the other allele. Sanger sequencing was performed with forward and reverse primers and each sequencing result was aligned to the GRCh38 human genome (see SMPD4 sequencing primer F and R, Supp Table 2). Alignment in Benchling confirmed a homozygous 43 base pair deletion in clone A8.C9 (C), which was used as our SMPD4 knockout (KO) line in the iPSC experiments in this manuscript.

Development • Supplementary information

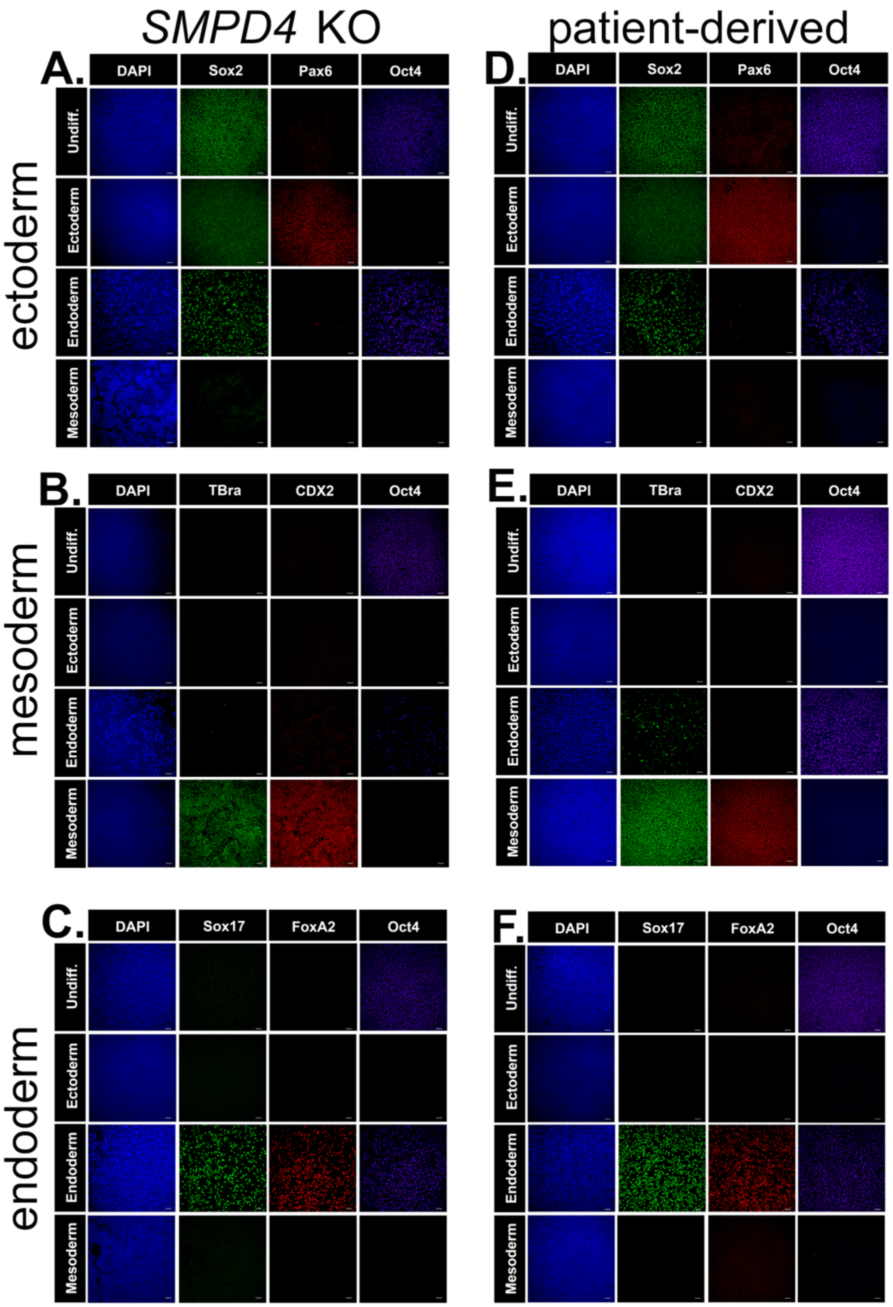

Development • Supplementary information

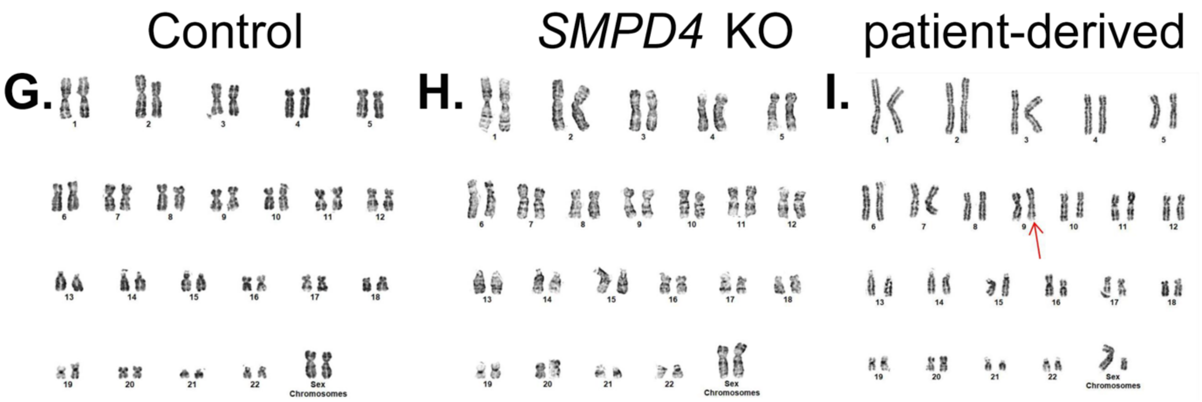

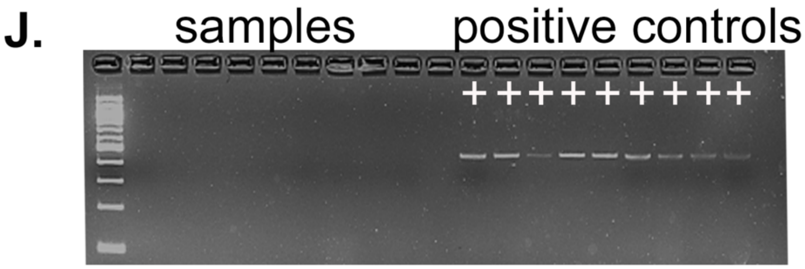

Fig. S9. Quality control of iPSC lines. (A-F) The *SMPD4KO* cell line was derived from the Control line so the trilineage differentiation capacity was tested for the *SMPD4KO* line (A-C) and patient derived line (D-F). Differentiation into ectoderm (A,D), mesoderm (B,E) and endoderm (C,F) following standard protocols generated cells with the expected immunoreactivity for each cell fate: SOX2 and PAX6 for ectoderm, TBra and CDX2 for mesoderm, and SOX17 and FOXA2 for endoderm. **(G-I)** Karyotyping show largely normal genomes in the control (G) and *SMPD4KO* (H) lines. We do note an inverted chromosome #9 for the *SMPD4* patient line (red arrow in I). This was not present in the initial patient isolate (data not shown) and therefore was likely acquired at some point in culture. Short tandem repeat profiles confirmed that the *SMPD4KO* line was from the control line, and these are distinct from the patient-derived cells. **(J)** A sample result from periodic mycoplasma testing in the lab showing all samples in this experiment (first eight lanes) were negative for mycoplasma contamination.

Development • Supplementary information

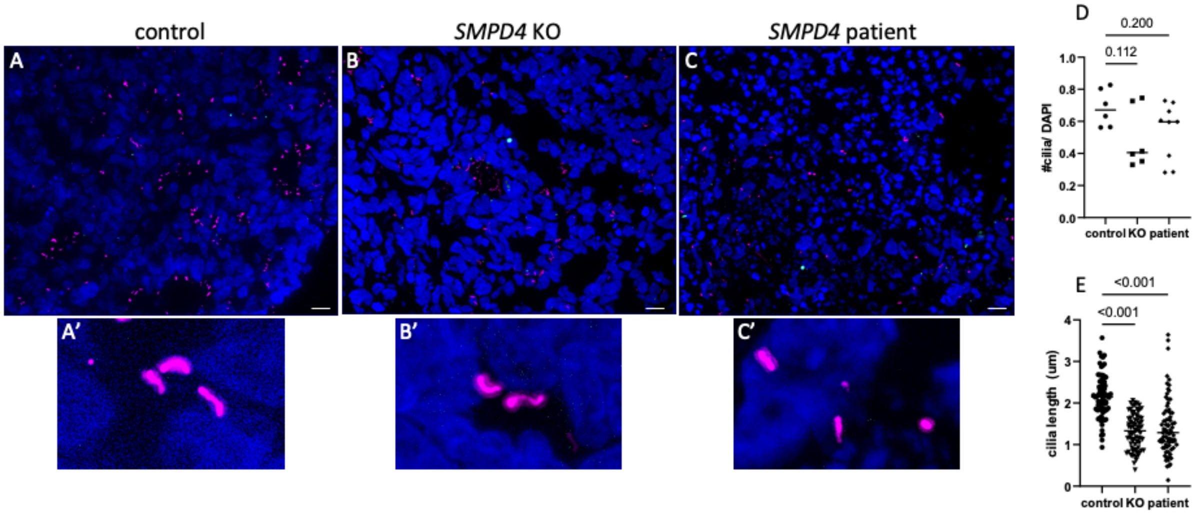

Fig. S10. Primary cilia are shortened in human iPSC-derived neural organoid *SMPD4* models. We generated three-dimensional neural organoids which model a later stage of human neural development than two-dimensional neural rosettes. At 28 days in vitro, control organoids exhibit widespread primary cilia lining each mini-ventricle (ARL13B in pink, A). *SMPD4* KO (B) and patient (C) iPSCs form neural organoids with abnormal cilia. Specifically, they do not have a significant decrease in the number of cilia per cell (n=6 control, 6 knockout and 9 patient, ANOVA p=0.144, D), but are shortened in length (n=75 control, 76 knockout and 77 patient, ANOVA p<0.0001, E). Scale bars in A-C = 10 μm.

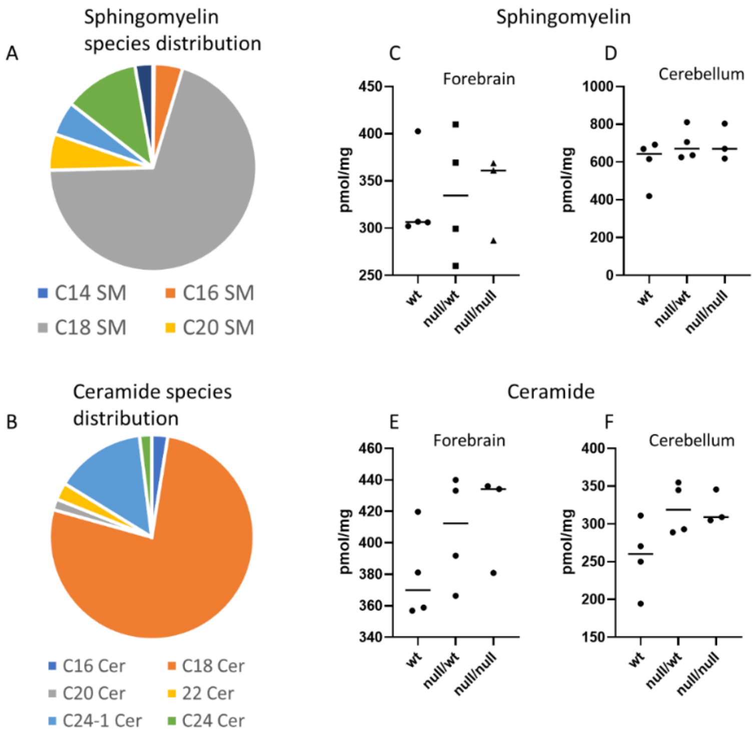

Fig. S11. Sphingomyelin and ceramide levels are unchanged in *Smpd4* null mouse brain. Relative amounts of sphingomyelin (A) and ceramide (B) species in control E18.5 embryos. Mass spectrometry of E18.5 mouse brain tissue for sphingomyelin and ceramide results are summarized in C,D and E,F respectively. No differences are seen in overall amount of either sphingolipid in forebrain (sphingomyelin n= 4 wild-type, 4 null/wt, 3 null/null, ANOVA p=0.975, C; ceramide n= 4 wild-type, 4 null/wt, 3 null/null, ANOVA p=0.302, E) or cerebellum (sphingomyelin n= 4 wild-type, 4 null/wt, 3 null/null, ANOVA p=0.377, D; ceramide n= 4 wild-type, 4 null/wt, 3 null/null, ANOVA p=0.079, F).

Development • Supplementary information

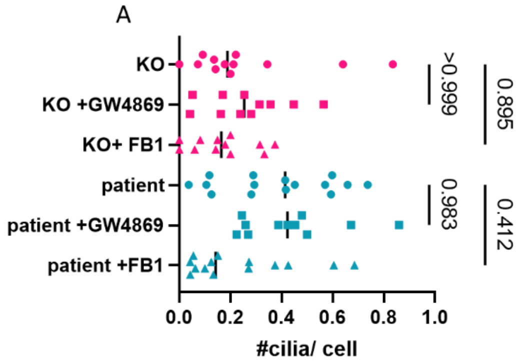

Fig. S12. *SMPD4* KO and patient iPSCs are unaffected by blocking neutral sphingomyelinase activity. *SMPD4* KO and patient lines do not have decreased cilia number with GW4869 or FB1 treatment. n=12 knockout, 11 GW4869 treated, 12 FB1 treated, ANOVA p=0.403; n= 15 patient, 11 GW4869 treated, 14 FB1 treated, ANOVA p=0.066.

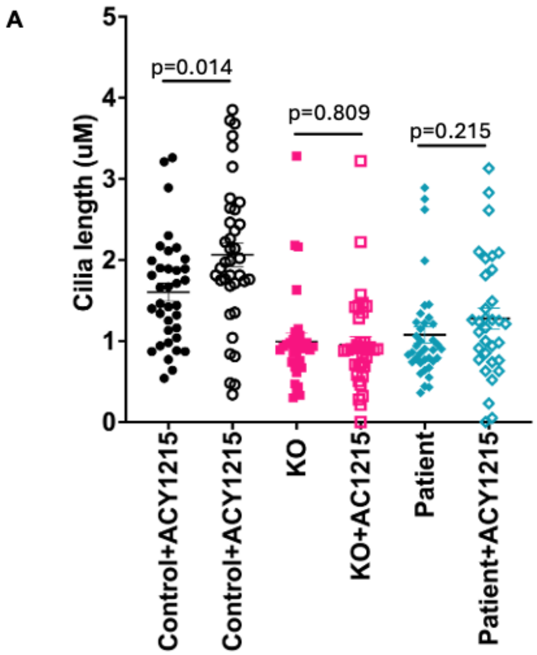

Fig. S13. Primary cilia in *SMPD4* KO and patient iPSCs do not lengthen in response to HDAC inhibition. Control cells do show longer primary cilia in response to ACY1215 treatment (n=36 control, 38 treated, p=0.01). Cilia in *SMPD4* KO (n=33 knockout, 36 treated, p=0.74) and patient lines (n=37 patient, 33 treated, p=0.21) do not see a similar increase in length.

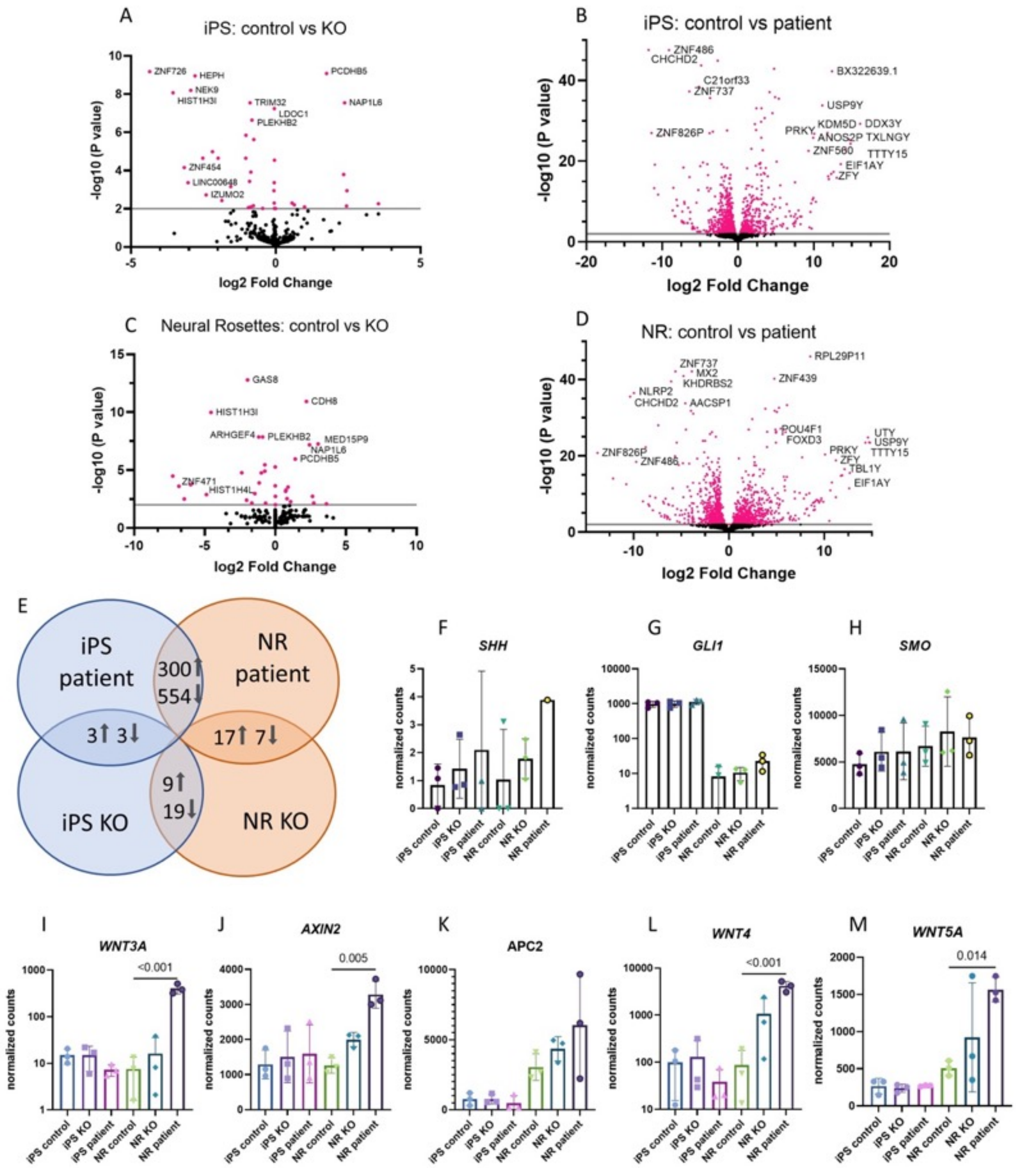

Fig. S14. Loss of *SMPD4* does not impact SHH signaling, but may deregulate the WNT pathway. Our RNA sequencing differential expression analysis showed that there were not many differentially expressed genes common between KO and patient datasets (A). We took a deeper look at SHH pathway genes and found SHH signaling appears unaffected by loss of *SMPD4.* The *SHH* ligand itself is expressed at low levels across all samples and is upregulated ~2 fold in patient neural rosette replicates only (B). *GLI1* and *SMO*, which are direct transcriptional targets of *SHH,* are unaffected (C,D). We also noted in our manual analysis that WNT signaling is upregulated. The central WNT pathway regulators *WNT3A* and *AXIN2* greatly increased in KO and patient NRs relative to control (I,J). We also identified upregulation of *APC2, WNT4,* and *WNT5A* (K-M).

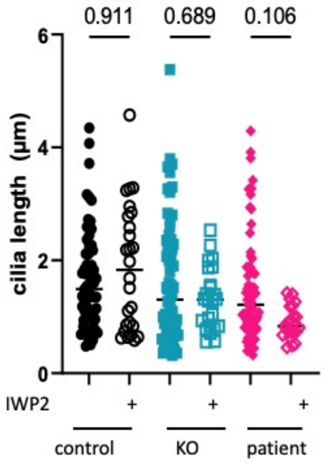

Fig. S15. Primary cilia in *SMPD4* KO and patient iPSCs do not lengthen in response to WNT inhibition. No cell population showed longer primary cilia in response to IWP2 treatment (n=65 control, 26 treated, p=0.911; n=63 knockout, 24 treated, p=0.689; n=80 patient, 22 treated, p=0.106).

Table S1. Mouse Lines

Mouse Line	Source	RRID	Abbreviation
Smpd4$^{tm2a(KOMP)Wtsi}$	**IMPC**	**MMRRC_062656-UCD**	***Smpd4***^{del}
Smpd4$^{tm2b(KOMP)Wtsi}$	**n/a**	**n/a**	***Smpd4***^{null}
Smpd4$^{tm2c(KOMP)Wtsi}$	**n/a**	**n/a**	***Smpd4***^{flox}
B6.FVB-Tg(EIIa-cre)$^{C5379Lmgd/J}$	**JAX #003724**	**IMSR_JAX:003724**	**EIIa-Cre**
FLP	**JAX # 012930**	**IMSR_JAX:012930**	**FLP**
B6.129S2-Emx1$^{tm1(cre)Krj/J}$	**JAX #005628**	**IMSR_JAX:005628**	**Emx1-Cre**
En1$^{tm2(cre)Wrst/J}$	**JAX #007916**	**IMSR_JAX:007916**	**En1-Cre**
B6.129T(SJL)-Foxg1$^{tm1.1(cre)Ddmo/J}$	**JAX #029690**	**IMSR_JAX:029690**	**Foxg1-Cre**
Smpd3 deletion	**CCHMC TAGE**	**n/a**	***Smpd3***^{del}

Table S2. CRISPR Guide Sequences and Genotyping Primers

Guide/Primer	Sequence
**Smpd3 Guide 1**	TGGCCAGAGCAGGCTGCACG CGG
**Smpd3 Guide 2**	CAGGTCCTAAAGCAGCAGTC AGG
**Smpd3 F1**	ACTGGATAAATGTTCAGCAGGTC
**Smpd3 F2**	AGGTTCCAGGAAGGCCAATTCAC
**Smpd3 R2**	TGCCTAGTAGGTGGGTAGAGGTC
**Smpd4 iPSC CRISPR guide 1**	AGCTTGATGACGACTTT
**Smpd4 iPSC CRISPR guide 2**	GACGCTATGGATCCCAC
**Smpd4 iPSC CRISPR guide 3**	GGAAGCCCCCAGGGCGC
**SMPD4 sequencing primer F**	TTCCTTGCTGGGGAGAAAGT
**SMPD4 sequencing primer R**	CTCGCCATTTCTCTTCCCCG
**Smpd4 WT F**	TGGTCCAATGATGAAGTTGG
**Smpd4 WT R**	GGGGGAAACTGGACTTTGTT
**Smpd4 transgene R**	GGCTAAAGGCTCACCAAAAA
**LacZ F**	GCTACCATTACCAGTTGGTCTGGTGTC
**Flox F**	AAGGCGCATAACGATACCAC
**Flox R**	CCGCCTACTGCGACTATAGAGA
**Cre F**	GCGGTCTGGCAGTAAAAACTATC
**Cre R**	GTGAAACAGCATTGCTGTCACTT
**En1-Cre F**	AGGCAAATTTTGGTGTACGG
**En1-Cre R**	GAGATTTGCTCCACCAGAGC
**Foxg1-Cre F**	TAGTGAAACAGGGGCAATGG
**Foxg1-Cre R**	TTCTCCCACATTGCACCTC

Development • Supplementary information

Table S3. Antibodies and Compounds

Antibody	Species	Source	RRID	IHC/ICC/Treatment Dilution
**ARL13B**	Anti-rabbit	Proteintech	AB_2060867	1:500
**ARL13B**	Anti-mouse	Neuromab.ucdavis	2877361	1:8000
**Brachyury (TBra)**	Anti-goat	R&D Systems	AF2085	1:500
**Calbindin**	Anti-rabbit	Abcam	AB_448597	1:2000
**Calnexin**	Anti-mouse	Thermo Fisher	AB_2069043	1:500
**CC3**	Anti-rabbit	CellSignaling	AB_2341188	1:300
**CDX2**	Anti-rabbit	Cell Marque	235R-14	1:500
**EdU**	n/a	Thermo Fisher	n/a	20 mg/kg
**FOXA2**	Anti-rabbit	Abcam	ab108422	1:500
**Gamma Tubulin**	Anti-mouse	Sigma Aldrich	AB_477584	1:2000
**GM130**	Anti-mouse	BD Transduction Labs	AB_398141	1:500
**Ki67**	Anti-rabbit	Abcam	AB_443209	1:200
**Lamin A/C**	Anti-mouse	Santa Cruz	AB_10991536	1:500
**NUP35**	Anti-rabbit	Sigma Aldrich	AB_1854720	1:300
**OCT4**	Anti-mouse	Santa Cruz	sc5279	1:500
**PAX6**	Anti-rabbit	MBL	AB_1520876	1:500
**PAX6**	Anti-rabbit	BioLegend	901301	1:500
**PHH3**	Anti-rabbit	Sigma Aldrich	AB_477043	1:500
**SMPD3**	Anti-rabbit	Abcam	n/a	1:200
**SMPD4**	Anti-rabbit	Sigma Aldrich	AB_2680762	1:200
**SOX2**	Anti-goat	R&D Systems	AF2018	1:500
**SOX17**	Anti-goat	R&D Systems	AF1924	1:500
**Ceramide (C16)**	n/a	Avanti Polar Lipids	n/a	2µM
**Fumonisin B1**	n/a	Sigma Aldrich	n/a	30µM
**GW4869**	n/a	Tocris Bioscience	n/a	10µM
**ACY1215**	n/a	ApexBio	n/a	1µM
**IWP2**	n/a	Tocris Bioscience	n/a	2µM

Table S4. *Smpd4 null* survival

	Total mice	wt/wt	null/wt	null/null	P value
**% expected**		25%	50%	25%	
**P21**	n=95	33%	59%	7%	<0.001
**P0**	n=43	35%	51%	14%	0.150

Table S5. *Smpd4 En1-Cre* conditional survival

	Total mice	no Cre; flox/wt	En1-Cre+; flox/wt	no Cre; null/flox	En1-Cre+; null/flox	p value
**% expected**		25%	25%	25%	25%	
**P21**	n=38	13%	24%	16%	13%	0.178
**P0**	n=46	33%	20%	20%	26%	0.695

Development • Supplementary information

Table S6. ***Smpd4 Emx1-Cre*** **conditional survival**

	Total mice	no Cre; flox/wt	Emx1-Cre+; flox/wt	no Cre; null/flox	Emx1-Cre+; null/flox	p value
**% expected**		25%	25%	25%	25%	
**P21**	n=38	13%	24%	16%	13%	0.178
**P0**	n=46	33%	20%	20%	26%	0.695

Table S7. ***Smpd4 Foxg1-Cre*** **conditional survival**

	Total mice	no Cre; flox/wt	Foxg1-Cre+; flox/wt	no Cre; null/flox	Foxg1-Cre+; null/flox	p value
**% expected**		25%	25%	25%	25%	
**P21**	n=24	33%	33%	13%	21%	0.370

Table S8. ***Smpd3*** **deletion survival (C57B6J)**

	Total mice	Smpd3 wt	Smpd3 del/wt	Smpd3 del/del	p value
**% expected**		25%	50%	25%	
**P21**	n=33	39%	61%	0%	0.003
**P0**	n=35	40%	60%	0%	0.002
**E18.5**	n=46	30%	59%	11%	0.086

Table S9. ***Smpd3*** **deletion survival (CD1)**

	Total mice	Smpd3 wt	Smpd3 del/wt	Smpd3 del/del	p value
**% expected**		25%	50%	25%	
**P0**	n=29	45%	55%	0%	0.003

Table S10. ***Smpd3; Smpd4*** **double knockout survival**

	Total mice	Smpd3$^{+/+}$; Smpd4$^{+/+}$	Smpd3$^{del/+}$; Smpd4$^{+/+}$	Smpd3$^{+/+}$; Smpd4$^{null/+}$	Smpd3$^{del/+}$; Smpd4$^{null/+}$	Smpd3$^{del/del}$; Smpd4$^{+/+}$	Smpd3$^{+/+}$; Smpd4$^{null/null}$	Smpd3$^{del/del}$; Smpd4$^{null/+}$	Smpd3$^{del/+}$; Smpd4$^{null/null}$	Smpd3$^{del/del}$; Smpd4$^{null/null}$	p value
**% expected**		6.25%	12.5%	12.5%	25%	6.25%	6.25%	12.5%	12.5%	6.25%	
**P21**	n=58	17%	17%	43%	17%	0%	3%	0%	2%	0%	<0.001
**E18.5-E16.5**	n=78	8%	14%	19%	26%	0%	8%	6%	17%	3%	0.001
**E14.5**	n=81	6%	12%	12%	31%	0%	9%	10%	15%	2%	0.355
