# Supplementary material for: SMPD4-mediated sphingolipid metabolism regulates brain and primary cilia development
Source: Development. 2024 Nov 15;151(22):dev202645. doi: 10.1242/dev.202645 (PMC11586524; doi:10.1242/dev.202645)
Supplement: Supplementary file 1 [file develop-151-202645_review_history.pdf]

SMPD4-mediated sphingolipid metabolism regulates brain and primary cilia development

Katherine Inskeep, Bryan Crase, Thamara Dayarathna and Rolf W Stottmann

DOI: 10.1242/dev.202645

Editor: Francois Guillemot
Review timeline
Original submission: 20 December 2023
Editorial decision: 26 January 2024
First revision received: 6 August 2024
Editorial decision: 12 September 2024
Second revision received: 15 October 2024
Accepted: 18 October 2024

Original submission

First decision letter

MS ID#: DEVELOP/2023/202645

MS TITLE: SMPD4 mediated sphingolipid metabolism regulates brain and primary cilia development

AUTHORS: Katherine Inskeep, Bryan Crase, and Rolf W Stottmann

I have now received the reports of three referees on your manuscript and I have reached a decision. The referees' comments are appended below, or you can access them online: please go to BenchPress and click on the 'Manuscripts with Decisions' queue in the Author Area.

As you will see, all the referees express great interest in your work, but they also have significant criticisms and recommend a substantial revision of your manuscript before we can consider publication. If you are able to revise the manuscript along the lines suggested, which may involve further experiments, I will be happy to receive a revised version of the manuscript. You should in particular consider addressing experimentally the point 1 of referee 2 regarding the causality between ciliary defects and brain abnormalities.

Your revised paper will be re-reviewed by one or more of the original referees, and acceptance of your manuscript will depend on your addressing satisfactorily the reviewers' major concerns. Please also note that Development will normally permit only one round of major revision. If it would be helpful, you are welcome to contact us to discuss your revision in greater detail. Please send us a point-by-point response indicating your plans for addressing the referees' comments, and we will look over this and provide further guidance.

Please attend to all of the reviewers' comments and ensure that you clearly highlight all changes made in the revised manuscript. Please avoid using 'Tracked changes' in Word files as these are lost in PDF conversion. I should be grateful if you would also provide a point-by-point response detailing how you have dealt with the points raised by the reviewers in the 'Response to Reviewers' box. If you do not agree with any of their criticisms or suggestions please explain clearly why this is so.

Reviewer 1

© 2024. Published by The Company of Biologists under the terms of the Creative Commons Attribution License (https://creativecommons.org/licenses/by/4.0/).

Advance summary and potential significance to field

In this manuscript, Inskeep et al. demonstrate that SMPD4, a sphingomyelinase that metabolizes sphingomyelin into ceramide, is essential for neural progenitor development, Purkinje cell survival/differentiation, and primary cilia length.
Variants in SMPD4 are associated with microcephaly, cerebellar hypoplasia, and ataxia. These studies, using cell type specific-mouse genetic models of Smpd3 and 4 deletion and human cortical organoids from SMDP4 patient iPSCs clearly demonstrate that SMPD4 is highly expressed in the developing cerebral cortex and cerebellum, affects NPC and cerebellar Purkinje cell survival, and is critical for the elaboration of a normal primary cilia. Further, the addition of exogenous ceremide rescues shortened primary cilia in SMPD4-deficient organoids. Bulk RNAseq analysis of organoids also hints at altered WNT signaling associated with ciliary defects. Together, these studies provide strong supportive evidence for SPMPD4's significance in NPC proliferation, cerebellar development, primary cilia maintenance, and how these functions may contribute to human microcephaly and cerebellar ataxic phenotypes. This is a fundamentally important observation that illuminates the essential nature of sphingomyelin biosynthesis mechanisms during brain development and highlights a potential cell biological pathway (i.e., primary cilia signaling) that may underly this function. The authors applied a robust multi-modal approach (e.g., comprehensive developmental mapping of Smpd3 and 4 expression, cell-type specific mouse genetic models of Smpd3 and 4 deletion patient-derived brain organoid models of SMPD4 disruption, and transcriptomic analysis of SMPD4 brain organoids [patient and KO]) in this well-designed study with high-quality data. As such this work is highly suitable for Development and makes a significant contribution to our understanding of cerebellar development primary cilia, and human brain disorders associated with disrupted sphingolipid biosynthesis. Furthermore, the well-documented collection of negative findings associated with global vs conditional deletion of SMPD3 and 4 in mice (vis-a-vis microcephaly; SFig. 3) adds valuable information. Overall, the manuscript is well-written, and the figures are clearly illustrated. Addressing the following points will be necessary to clarify and strengthen the main aspects of this work.

Comments for the author

SMPD4 deficient organoids also have shortened, dysmorphic primary cilia- they tend to have bulbous distal tips. Are they shedding ciliary vesicles and undergoing ectosis? This issue should be addressed in the discussion as it may indicate a ciliary signaling defect.

Does SMPD4 play different roles in cilia formation/maintenance in mice vs humans?-
-SMPD4 KO and patient organoids have shortened primary cilia whereas En1-Cre+; Smpd4flox/null Purkinje cell cilia are significantly longer.

Additional discussion about why the authors think primary cilia changes are what drives cerebellar developmental changes in SMPD4 deficient mice and humans, not the other way around (i.e., changes in primary cilia morphology are a byproduct of changes in cell survival/ differentiation following SMPD4 deletion) will be helpful.

Reviewer 2

Advance summary and potential significance to field

In their manuscript, Inskeep et al. investigate the role of sphingolipid metabolism in brain development by analysing the function of the SMPD4 gene, mutations in which can lead to microcephaly and cerebellar hypoplasia. First, they showed that unlike human patients Smpd4 mutant mice did not develop a smaller brain and ruled out redundancy with another neutral sphingomyelinase, Smpd3, as a potential reason for the absence of a microcephaly phenotype. Smpd4 mutant mice though had a smaller cerebellum due to increased apoptosis of Purkinje neurons. In contrast, analysis of 2D neural cultures as well as 3D organoid models derived from either patient or CRISPR/Cas9 induced knock-out iPSCs revealed increased cell death of neural progenitors. Interestingly, primary cilia were longer and shorter in mouse Purkinje neurons and in

© 2024. Published by The Company of Biologists under the terms of the Creative Commons Attribution License (https://creativecommons.org/licenses/by/4.0/).

human neural progenitors, respectively. Finally, ciliary length could be rescued by adding exogenous ceramide in the human models.

Taken together, the authors show that SMPD4 mediated generation of ceramide is critical for the development of primary cilia and the brain in humans and in mice. They use a valid combination of murine and human model systems to address the largely unexplored role of sphingolipid metabolism on brain development. Elucidating such functions is important given the severe consequences of SMPD4 mutations on human brain development, The manuscript is well written and the authors present convincing data, however, there are several shortcomings the authors need to address before publication.

Comments for the author

Major points:
1. The authors convincingly show that SMPD4 mutations lead to brain malformations and affect ciliary length in mouse and human models but it remains unclear whether the ciliary defects correlate with or are causative of the brain abnormalities. The authors extensively discuss potential widespread roles of ceramide raising the possibility of cilia independent functions in the development of the SMPD4 phenotypes. To address this issue, the authors could treat neural rosettes with HDAC6 inhibitors to restore cilia length with the aim to rescue proliferation defects in neural progenitors. It is also puzzling that the mutation has opposing effects on ciliary length in Purkinje neurons and in neural progenitor cells. The authors claim that cilia in the human models were dysmorphic and bulbous but this cannot be seen very well in the provided images. This type of statement requires careful electron microscopy analysis.

2. How the changes in ciliary length affect signalling pathways and thereby cause Purkinje cell death and reduced proliferation in neural rosettes remains largely unknown. The authors claim enhanced Wnt signalling in mutant neural rosettes but no experimental data is provided to support this statement (it looks like data in Supplementary Figure 12 I-M is missing). In this respect, how did they obtain this finding and what does manual analysis mean? Have the supposed changes in gene expression been validated by qRT-PCR? Does blocking Wnt signalling rescue the decreased proliferation?

3. It also remains unknown whether the loss of Purkinje neurons is linked to the reduced proliferation or premature differentiation of GPCs. It is not quite clear which defect proceeds the other. The facts that Purkinje cells are sources of Shh for GPCs and SMPD4 mutations affect ciliary length in other neural cell types raise the intriguing possibility that cilia and Shh signalling are affected in GPCs.
4. The number of mutant iPSC lines is at the very low end of the spectrum. Importantly, no control patient line was used raising doubts about the reproducibility of the data derived from 2D cultures and 3D organoids. This is particularly obvious with the RNAseq data which appears to be based on n=1 biological replicate for the controls.

5. A more careful description of the SMPD4 expression pattern would be beneficial to better judge SMBD4 functions. In the developing cortex, it looks like it shows a graded expression within the VZ. Is it expression confined to Pax6+ radial glial cells or does it extend to basal progenitors and neurons? In the developing cerebellum, is Smpd4 expressed in granule precursor cells and neurons?

Minor points:

1. The authors need to be more careful when assigning significant changes. This should only be done when $p<0.05$ and affects conclusions on the survival of Smbd4 mutants (Fig. 2A), latency on the rotarod (Fig. 3T), rosette area (Fig. 4E) and size of KO organoids (Fig. 4I).

2. Smpd3 knock-out: the authors report no other appreciable deletion on chromosome 8, but Supp Fig. 7B suggests additional chromosomal abnormalities throughout the genome. Are the coding regions of other genes affected?

3. The brightfield images in Figure 4A are of poor quality, rosettes and cells are hardly visible.

© 2024. Published by The Company of Biologists under the terms of the Creative Commons Attribution License (https://creativecommons.org/licenses/by/4.0/).

4. The authors conclude that “because fewer NPCs are present, proliferate less, and fail to survive, many fewer total neurons are produced” but they did not determine neuron numbers in their organoid models.

5. Cilia are hardly visible in Purkinje cells. It looks like CALBINDIN and ARL13B are visualized in the same colour.

6. Page 7: It should read Fig. 5I-M

7. Supplementary Figures 9 and 10 seem to have been swapped.

Reviewer 3

Advance summary and potential significance to field

The goal of this study is to investigate how SMPD4 (and Smpd3) contribute to early brain development. Mutations in SMPD4 cause a developmental disorder associated with microcephaly and other neurological alterations. The authors use mouse models and human cellular models to determine how mutations in SMPD4 affect the proliferation of progenitor cells and the survival of newborn neurons. They also observe alterations in cilia production that can be reversed by supplementation with ceramides. The advances made by this study are the further characterizations of mouse lines with Smpd4 and Smpd3 mutations as well as the generation of human cellular models with SMPD4 mutations. Using these models, the authors show that the mutations impact both human and mouse brain development, albeit with some species specific effects. They also suggest that defective cilia may contribute to the developmental alterations observed in SMPD4 disorder.

Comments for the author

The strengths of the study are the use of both genetic mouse and human cellular systems, as well as the multiple different analyses applied to these models.
Together the experiments are comprehensive, incorporating several different types of models and techniques. The introduction and discussion are clear and well-written and the rationale for the study is clearly presented.

That said, there are also some significant weaknesses. In general, while the paper makes a lot of different observations, these are not really brought together into a central conclusion or clear mechanistic understanding. In addition, I feel that some of the conclusions need additional support (see specific comments below). Going deeper to substantiate some of the main findings would strengthen the paper. In terms of technical aspects, there needs to be more validation of the stem cell models, in particular quality control data. It is concerning that there is no isogenic control for the patient iPSC line. With only one patient line and one unrelated control line, there can be many differences driven by stem cell line/genetic background that are unrelated to the disease. The authors could consider only using the isogenic control line together with the isogenic gene-edited KO line.

Specific points:
- Quality control data should be provided for the stem cells used (e.g. genomic integrity and pluripotency status)
-The figure legends should include information about the statistical tests used and sample size.
-The analysis of SMPD4 subcellular localization in HEK293T cells doesn’t seem relevant as the protein may have a very different expression pattern in neural progenitors and different types of neurons. These results also presented qualitatively with just a few example images. If these conclusions are important for the paper, perhaps they should be revisited in relevant neural cell types with quantitative assays.
-What cell type is in the KO organoids at d28 if it’s not NPCs? The authors should stain for other cell type markers (e.g. other neural progenitor types neurons, etc)

© 2024. Published by The Company of Biologists under the terms of the Creative Commons Attribution License (https://creativecommons.org/licenses/by/4.0/).

-The differential effects on cilia in the two systems (mouse vs human) is interesting. However, it's hard to directly compare them as in the mouse postnatal Purkinje Cells are examined but in the human cellular models it's an earlier developmental stage and a different cell type. Perhaps this should be discussed.
-The ceramide levels shown in Sup. Fig. 8 do appear to be increasing in the mutants. This likely doesn't reach statistical significant due to the small sample size and high variability. This is a key experiment that should be repeated with more samples to conclude whether there is an effect or not. --
Related to this, the authors could consider doing the sphingomyelin & ceramide quantification experiments in the human cell system where greater defects were observed. This seems important to justify the ceramide supplementation experiments. e.g. Is the "rescue" caused by reversal of ceramide deficiency or by boosting ceramide levels above normal?
-The manuscript ends in a rather inconclusive point with the RNAseq results. It is somewhat unclear how these results tie in with the rest of the story.
-The statement in discussion that we "propose a novel role for SMPD4 in controlling ceramide distribution to the nuclear envelope and primary cilium…"
seems a bit overstated.
-In the discussion, the authors suggest that bulk ceramide supplementation may not alter local concentration in a therapeutic way; however, they do the supplementation experiment and show that it can impact cilia morphology. These seem like contradictory viewpoints.
-In Fig 1K, Smpd4 expression appears to be in granule cells, not PCs, is this correct?
-In Fig. 2L,M the authors measure area but it seems that brain volume or weight would be better metrics for measuring brain/brain region size.
-Fig. 3 – It would be helpful to show example images for all stainings (e.g. Pax6, PHH3, etc)
-In Fig. 3T and U, are Control and null mice statistically different with post-hoc testing? What statistical test was done here?
-In Fig. 4B-D, DAPI or other nuclei stain should be shown to see the cell density and rosette architecture.
-Fig. 5E,F – is the ARL13B+ staining in magenta? If so, is it expected to stain the whole cell body? Are the "dots" of magenta the cilia? This should perhaps be verified with an additional cilia marker. From the example images is does not look like these "dots" are longer in the mutant tissue.
-For the inhibitor treatments in Fig. 6, is it possible that they are having a general non-specific effect on cell health, rather than regulating cilia production, specifically? For example, are treatments causing death of non-ciliated cells?

Minor comments/suggestions:

-Is anything known about cilia from post-mortem patient specimens that would lend face validity to the findings reported here? (It's understood that these may not be available).
-It would be helpful to know what other brain regions Smpd4 is expressed in besides the cortex and cerebellum.
-It would be preferable to see the mRNA expression at 12.5, rather than stating data not shown.
-Perhaps Fig. 1M should come first before discussing brain localization?
-What about cerebellar development in the Smpd3-del mice? Perhaps the statement "dispensable for brain development" should be softened since other aspects of development could be altered, which were not looked at.
-When describing Fig. 5A-D results, please indicate which mouse line the cultures were from.
-On page 7, the figure number is missing (it says "Fig I-M")
-On page 8 of the results, it would be helpful to indicate what GW4869 and FB1 are (although this is shown in the figure, it would be helpful to have it in the results as well).
-Scale bars should be shown for all images in the supplementary figures (and also Fig. 2J, K).
-It is unclear what the "Quit fraction" label indicates on Sup Fig. 2.

First revision

Author response to reviewers' comments

© 2024. Published by The Company of Biologists under the terms of the Creative Commons Attribution License (https://creativecommons.org/licenses/by/4.0/).

Reviewer 1 Advance Summary and Potential Significance to Field:
In this manuscript, Inskeep et al. demonstrate that SMPD4, a sphingomyelinase that metabolizes sphingomyelin into ceramide, is essential for neural progenitor development, Purkinje cell survival/differentiation, and primary cilia length.
Variants in SMPD4 are associated with microcephaly, cerebellar hypoplasia, and ataxia. These studies, using cell type specific-mouse genetic models of Smpd3 and 4 deletion and human cortical organoids from SMDP4 patient iPSCs clearly demonstrate that SMPD4 is highly expressed in the developing cerebral cortex and cerebellum, affects NPC and cerebellar Purkinje cell survival, and is critical for the elaboration of a normal primary cilia. Further, the addition of exogenous ceremide rescues shortened primary cilia in SMPD4-deficient organoids. Bulk RNAseq analysis of organoids also hints at altered WNT signaling associated with ciliary defects. Together, these studies provide strong supportive evidence for SPMPD4's significance in NPC proliferation, cerebellar development, primary cilia maintenance, and how these functions may contribute to human microcephaly and cerebellar ataxic phenotypes. This is a fundamentally important observation that illuminates the essential nature of sphingomyelin biosynthesis mechanisms during brain development and highlights a potential cell biological pathway (i.e., primary cilia signaling) that may underly this function. The authors applied a robust multi-modal approach (e.g., comprehensive developmental mapping of Smpd3 and 4 expression, cell-type specific mouse genetic models of Smpd3 and 4 deletion, patient-derived brain organoid models of SMPD4 disruption, and transcriptomic analysis of SMPD4 brain organoids [patient and KO]) in this well-designed study with high-quality data. As such this work is highly suitable for Development and makes a significant contribution to our understanding of cerebellar development, primary cilia, and human brain disorders associated with disrupted sphingolipid biosynthesis. Furthermore, the well-documented collection of negative findings associated with global vs conditional deletion of SMPD3 and 4 in mice (vis-a-vis microcephaly; SFig. 3) adds valuable information. Overall, the manuscript is well- written, and the figures are clearly illustrated. Addressing the following points will be necessary to clarify and strengthen the main aspects of this work.

Reviewer 1 Comments for the Author:
SMPD4 deficient organoids also have shortened, dysmorphic primary cilia- they tend to have bulbous distal tips. Are they shedding ciliary vesicles and undergoing ectosis? This issue should be addressed in the discussion as it may indicate a ciliary signaling defect.

We have added this to a revised discussion as part of a new paragraph.

"We also show evidence for disrupted primary ciliary morphology in this study. We note changes in length in multiple settings upon loss of SMPD4. We also observed that the cilia in organoids sometimes have a anomalous bulbous tip. This has previously been associated with accumulation of cargo at the distal tip of the cilia secondary to disrupted retrograde ciliary transport (Tran et al., Nat Genetics). An alternative explanation may be a shedding of ciliary vesicles and ectocytosis which potentially affects ciliary signaling. This is a process more recently appreciated in ciliary biology (e.g., PMID 28017328; 34095126, 34533135, 36222105) and may be a fruitful topic of further exploration in sphingomyelinase biology."

Does SMPD4 play different roles in cilia formation/maintenance in mice vs humans?--SMPD4 KO and patient organoids have shortened primary cilia whereas En1-Cre+; Smpd4flox/null Purkinje cell cilia are significantly longer.

Additional discussion about why the authors think primary cilia changes are what drives cerebellar developmental changes in SMPD4 deficient mice and humans, not the other way around (i.e., changes in primary cilia morphology are a byproduct of changes in cell survival/ differentiation following SMPD4 deletion) will be helpful.

We have address this in a revised discussion as part of the same new paragraph.
"We espouse a model where the cell intrinsic effects of SMPD4 deficiency lead to primary cilia defects which then, in turn, at least contribute to the changes in the affected tissues. A different model could suppose that SMPD4 deficiency alters the tissue and the effects on cilia are in response to this primary insult. While this is a perfectly valid model and might help explain the species specific effects on cilia we observe in our different models, we prefer the first model at this time given the large body of evidence that primary cilia drive growth and differentiation

© 2024. Published by The Company of Biologists under the terms of the Creative Commons Attribution License (https://creativecommons.org/licenses/by/4.0/).

of the developing nervous system."

*****

Reviewer 2 Advance Summary and Potential Significance to Field:
In their manuscript, Inskeep et al. investigate the role of sphingolipid metabolism in brain development by analysing the function of the SMPD4 gene, mutations in which can lead to microcephaly and cerebellar hypoplasia. First, they showed that unlike human patients Smpd4 mutant mice did not develop a smaller brain and ruled out redundancy with another neutral sphingomyelinase, Smpd3, as a potential reason for the absence of a microcephaly phenotype. Smpd4 mutant mice though had a smaller cerebellum due to increased apoptosis of Purkinje neurons. In contrast, analysis of 2D neural cultures as well as 3D organoid models derived from either patient or CRISPR/Cas9 induced knock-out iPSCs revealed increased cell death of neural progenitors. Interestingly, primary cilia were longer and shorter in mouse Purkinje neurons and in human neural progenitors, respectively. Finally, ciliary length could be rescued by adding exogenous ceramide in the human models.

Taken together, the authors show that SMPD4 mediated generation of ceramide is critical for the development of primary cilia and the brain in humans and in mice. They use a valid combination of murine and human model systems to address the largely unexplored role of sphingolipid metabolism on brain development. Elucidating such functions is important given the severe consequences of SMPD4 mutations on human brain development, The manuscript is well written and the authors present convincing data, however, there are several shortcomings the authors need to address before publication.

Reviewer 2 Comments for the Author:
Major points:

1.The authors convincingly show that SMPD4 mutations lead to brain malformations and affect ciliary length in mouse and human models but it remains unclear whether the ciliary defects correlate with or are causative of the brain abnormalities. The authors extensively discuss potential widespread roles of ceramide raising the possibility of cilia independent functions in the development of the SMPD4 phenotypes. To address this issue, the authors could treat neural rosettes with HDAC6 inhibitors to restore cilia length with the aim to rescue proliferation defects in neural progenitors. It is also puzzling that the mutation has opposing effects on ciliary length in Purkinje neurons and in neural progenitor cells. The authors claim that cilia in the human models were dysmorphic and bulbous but this cannot be seen very well in the provided images. This type of statement requires careful electron microscopy analysis.

We have followed this suggestion and treated the neural rosettes with HDAC6 inhibitors and test if cilia length is restored along with proliferation rates. We did indeed see an increase in cilia length in control rosettes but not in the patient-derived or knockout rosettes. Given the cilia did not lengthen, we did not address a rescue in proliferation. This experiment is shown in a new supplemental figure 13.

Bulbous cilia have often been shown in the literature without resorting to electron microscopy. We have revisited our data and chosen new figures to place in the inset panels in figure 5 to more clearly demonstrate this finding. As this is a relatively minor point, we do not favor the use of considerable resources to demonstrate this with electron microscopy.

2.How the changes in ciliary length affect signalling pathways and thereby cause Purkinje cell death and reduced proliferation in neural rosettes remains largely unknown. The authors claim enhanced Wnt signalling in mutant neural rosettes but no experimental data is provided to support this statement (it looks like data in Supplementary Figure 12 I-M is missing). In this respect, how did they obtain this finding and what does manual analysis mean? Have the supposed changes in gene expression been validated by qRT-PCR? Does blocking Wnt signalling rescue the decreased proliferation?

We have included the bar graphs in (now 14 I-M) in our revision and recognize this was a particularly unfortunate omission from the previous data set. These changes were identified during a manual review of the normalized RNA- Seq result table for lists of genes associated with

© 2024. Published by The Company of Biologists under the terms of the Creative Commons Attribution License (https://creativecommons.org/licenses/by/4.0/).

several signaling pathways (i.e., candidate gene approach), including Shh and Wnt, and pulled out any that had signal above baseline. These were not further validated by qRT- PCR and we are happy to discuss why we think RNA-Seq in triplicate is a superior measure of transcriptional differences. We did treat the iPSCs with a WNT inhibitor but this did not appear to rescue cilia length. We did include the results of this fairly minimal experiment as a new supplemental figure 13, although this experiment is not especially well powered.

3.It also remains unknown whether the loss of Purkinje neurons is linked to the reduced proliferation or premature differentiation of GPCs. It is not quite clear which defect proceeds the other. The facts that Purkinje cells are sources of Shh for GPCs and SMPD4 mutations affect ciliary length in other neural cell types raise the intriguing possibility that cilia and Shh signalling are affected in GPCs.

We agree this is an interesting question to pursue in potential future studies and this was a model we considered. We show data that EGL PAX6-positive and pHH3-positive cells are not changed suggesting that there is no defect in GCP proliferation at this developmental stage. We have measured cilia number and length in mouse GCPs in section immunohistochemistry in a preliminary experiment and did not observe any difference in cilia in these cell types. We also measured the number of GCPs in E18.5 mouse cerebellum by staining for PAX6. While we did find a slight difference in the ratio of migrating GCPs in the forming inner granule layer to GCPs remaining in a proliferative state in the external granule layer, there was no overall change in the number of GCPs present per area.

4.The number of mutant iPSC lines is at the very low end of the spectrum. Importantly, no control patient line was used raising doubts about the reproducibility of the data derived from 2D cultures and 3D organoids. This is particularly obvious with the RNAseq data which appears to be based on n=1 biological replicate for the controls.

The RNASeq data was performed in triplicate (n=3) for all three conditions, including control. We have made this explicit in the materials and methods. We were not able to use an isogenic control for the patient derived cell. We did not get cells from another family member (still would not be truly isogenic) and a world class Genome editing core at Cincinnati Childrens Hospital was not able to edit the patient cells back to reference sequence with many months of effort. This is why we resorted to a KO cell line, but we recognize the limitations of this experiment.

5.A more careful description of the SMPD4 expression pattern would be beneficial to better judge SMBD4 functions. In the developing cortex, it looks like it shows a graded expression within the VZ. Is it expression confined to Pax6+ radial glial cells or does it extend to basal progenitors and neurons? In the developing cerebellum, is Smpd4 expressed in granule precursor cells and neurons?

We indeed have data to address this point and have significantly revised Figure 1 in response to this comment. Expression is not confined to Pax6+ cells and it does extend into the Tbr2+ and Tuj1+ layers in the cortex. We show multiple stages of cerebellar development where the Smpd4 expression is similarly broad.

Minor points:

1.The authors need to be more careful when assigning significant changes. This should only be done when $p<0.05$ and affects conclusions on the survival of Smbd4 mutants (Fig. 2A), latency on the rotarod (Fig. 3T), rosette area (Fig. 4E) and size of KO organoids (Fig. 4I).

We no longer prefer to "assign" significant changes to the data. We resist the commonly held principle of assigning significance to an experiment with a p-value of 0.04999 but not one with 0.051. This is accordance with a more nuanced approach to statistical analysis of biological data as espoused by the American Statistical Association (Yaddanapudi J Anaesthesiol Clin Pharmacol. 2016 Oct-Dec; 32(4): 421–423) and others. We have performed experiments with appropriate statistical power and present all p-values. We limit our use of the word significant and empower the reader to draw their own conclusions as we present the data. In light of the reviewer's valid

© 2024. Published by The Company of Biologists under the terms of the Creative Commons Attribution License (https://creativecommons.org/licenses/by/4.0/).

concern, we have reviewed our description of these data sets mentioned to ensure we have not over-interpreted our data, or used the term "significant" inappropriately and revised with these comments in mind.

The P0 survival data presentation (p=0.150) in the revision is not described as significant but the P21 data (p=0.0004) is. (We believe this difference is simply due to sample sizes at P0 vs P21 but refrained from collecting and sacrificing more animals simply for the value of the p-value statistic). The rotarod data was only described as the mice "performed worse… with shorter latency to fall" and the p-value was presented in 3T as p=0.055. Similarly, we described the rosette area in Fig 4 as "they have a smaller area and diameter" with a p-value of 0.053.

2.Smpd3 knock-out: the authors report no other appreciable deletion on chromosome 8, but Supp Fig. 7B suggests additional chromosomal abnormalities throughout the genome. Are the coding regions of other genes affected?

We present an entire list of deletions in the spirit of full disclosure (Supp Figure 7). As none of these are on the same chromosome as Smpd3, they are almost certainly not genetically linked to Smpd3 and will not have a consistent bearing on any phenotypes we report.

3.The brightfield images in Figure 4A are of poor quality, rosettes and cells are hardly visible.

We have replaced all of these with improved pictures.

4.The authors conclude that "because fewer NPCs are present, proliferate less, and fail to survive, many fewer total neurons are produced" but they did not determine neuron numbers in their organoid models.

We derive this conclusion from the markedly smaller structures produced and appreciable in the presented pictures.

5.Cilia are hardly visible in Purkinje cells. It looks like CALBINDIN and ARL13B are visualized in the same colour.

This is true as both antibodies are in rabbit. We had initially thought these antibodies and structures were so well validated and known to the field, this issue would not come up. However, we did not present our data in the best light given this. We have collated our data and improved the presentation of this finding in a revised Fig 5.

6.Page 7: It should read Fig. 5I-M

Thanks - this appropriately revised.

7.Supplementary Figures 9 and 10 seem to have been swapped.
Thanks - supplementary figures have been revised.

*****
Reviewer 3 Advance Summary and Potential Significance to Field:
The goal of this study is to investigate how SMPD4 (and Smpd3) contribute to early brain development. Mutations in SMPD4 cause a developmental disorder associated with microcephaly and other neurological alterations. The authors use mouse models and human cellular models to determine how mutations in SMPD4 affect the proliferation of progenitor cells and the survival of newborn neurons. They also observe alterations in cilia production that can be reversed by supplementation with ceramides. The advances made by this study are the further characterizations of mouse lines with Smpd4 and Smpd3 mutations as well as the generation of human cellular models with SMPD4 mutations. Using these models, the authors show that the mutations impact both human and mouse brain development, albeit with some species specific effects. They also suggest that defective cilia may contribute to the developmental alterations observed in SMPD4 disorder.

Reviewer 3 Comments for the Author:

© 2024. Published by The Company of Biologists under the terms of the Creative Commons Attribution License (https://creativecommons.org/licenses/by/4.0/).

The strengths of the study are the use of both genetic mouse and human cellular systems, as well as the multiple different analyses applied to these models. Together the experiments are comprehensive, incorporating several different types of models and techniques. The introduction and discussion are clear and well-written and the rationale for the study is clearly presented.

That said, there are also some significant weaknesses. In general, while the paper makes a lot of different observations, these are not really brought together into a central conclusion or clear mechanistic understanding. In addition, I feel that some of the conclusions need additional support (see specific comments below). Going deeper to substantiate some of the main findings would strengthen the paper. In terms of technical aspects, there needs to be more validation of the stem cell models, in particular quality control data. It is concerning that there is no isogenic control for the patient iPSC line. With only one patient line and one unrelated control line, there can be many differences driven by stem cell line/genetic background that are unrelated to the disease. The authors could consider only using the isogenic control line together with the isogenic gene-edited KO line.

We agree this last point is valid and have described elsewhere the inability to generate an isogenic control for the patient line due to unknown technical factors. We think the patient line has value in concert with the control/KO comparison but recognize the caveats raised by the reviewers. In reviewing the data, the control vs KO comparisons alone would illustrate most, if not all, of the points we are making. However, with the data from the patient-derived cells in hand, we feel like this is worth presenting while fully aware of the caveats raised. We hope this nuance is better expressed in this revision.

Specific points:
- Quality control data should be provided for the stem cells used (e.g. genomic integrity and pluripotency status).

This is now included as Supplemental Figure 9. We have performed multiple analyses on these cells. Karyotyping, STR DNA profiling, mycoplasma testing results, and trilineage differentiation potential are now all reported. This was done at the world class stem cell core at Cincinnati Children's Hospital Medical Center. Coordinating, completing and data analysis of these experiments comprise the bulk of the delay in preparation of this manuscript revision.

-The figure legends should include information about the statistical tests used and sample size.

These have been edited in this way. More information about statistical tests is now found in the Materials and Methods as well.

-The analysis of SMPD4 subcellular localization in HEK293T cells doesn't seem relevant as the protein may have a very different expression pattern in neural progenitors and different types of neurons. These results also presented qualitatively with just a few example images. If these conclusions are important for the paper, perhaps they should be revisited in relevant neural cell types with quantitative assays.

We understand this concern about cell type relevance. We demonstrated this qualitative analysis mostly in the spirit of comparison to SMPD3 as part of our discussion about potential compensation between these two genes with similar enzyme activity. We began to address this concern/idea raised by the reviewer and found the SMPD4 antibody has been less successful in other cell lines so we are not confident we would get robust data.

-What cell type is in the KO organoids at d28 if it's not NPCs? The authors should stain for other cell type markers (e.g. other neural progenitor types, neurons, etc)

At this stage, the organoids would consist of primarily neurons, along with other cell types that would be present at a similar stage in human brains in vivo. We are confident this has been robustly addressed in other literature in the field.

-The differential effects on cilia in the two systems (mouse vs human) is interesting. However,

© 2024. Published by The Company of Biologists under the terms of the Creative Commons Attribution License (https://creativecommons.org/licenses/by/4.0/).

it's hard to directly compare them as in the mouse, postnatal Purkinje Cells are examined but in the human cellular models it's an earlier developmental stage and a different cell type. Perhaps this should be discussed.

We recognize and agree with the reviewer's point. Some initial thoughts are presented below. We add a more succinct version of this in the revised discussion.

We believe the benefit of utilizing both model systems is to robustly demonstrate the role of this gene while overcoming experimental and/or biological hurdles that complicate assessment in a given organism. For example, major components of cerebellar development including granule cell proliferation and Purkinje cell dendritic arbor expansion happen primarily in the early postnatal period, P0-P21 in mice and Year 0-21 in humans. We cannot feasibly study human postnatal cerebellar development using a human iPSC-derived brain organoid system as this technology has not yet been developed. Therefore we've used the best available alternative. Mouse and human cerebellum share many key developmental pathways, and the cerebellum is one of the most ancestral brain regions between these species. Although the role of primary cilia in cerebellar development has been studied in several robust publications, the reason for this organelle's apparent distinct roles in mouse vs human brain has not yet been elucidated but would be interesting as a topic for future study.

-The ceramide levels shown in Sup. Fig. 8 do appear to be increasing in the mutants. This likely doesn't reach statistical significant due to the small sample size and high variability. This is a key experiment that should be repeated with more samples to conclude whether there is an effect or not. -- Related to this, the authors could consider doing the sphingomyelin & ceramide quantification experiments in the human cell system where greater defects were observed. This seems important to justify the ceramide supplementation experiments. e.g. Is the "rescue" caused by reversal of ceramide deficiency or by boosting ceramide levels above normal?

We initially shared all of these concerns and sentiments. We also noted the variability in the results when we received them. The director of the mass spectrometry core who guided these experiments is a true expert in this area of biology and felt that further experiments were NOT likely to yield significantly different results. They also thought results from the native tissue would be much more informative than a parallel experiment from in vitro tissues. We understand the concern but have deferred to the content expert in this area.

-The manuscript ends in a rather inconclusive point with the RNAseq results. It is somewhat unclear how these results tie in with the rest of the story.

We have discussed this further. We attempted to identify a transcriptional signature to the loss of SMPD4 and did not see such a thing. This is another "negative" result, but we think it is worth sharing. There are also some predictions that could be made as a consequence of the ciliary disruptions we show that are not borne out in these RNASeq experiments. We think that ciliary experts would be interested to see this relatively unimposing transcriptional response, or at least know we considered the potential implications.

The statement in discussion that we "propose a novel role for SMPD4 in controlling ceramide distribution to the nuclear envelope and primary cilium…" seems a bit overstated.

We have revised this statement in deference to this comment.

-In the discussion, the authors suggest that bulk ceramide supplementation may not alter local concentration in a therapeutic way; however, they do the supplementation experiment and show that it can impact cilia morphology. These seem like contradictory viewpoints.

This is a fair point and our observation was not initially well-written. The supplementation experiment performed was done on iPS cells in monolayer culture. We didn't have to contend with blood-brain barrier, uptake on a tissue level, etc. We think it would be a lot harder to show therapeutic effect of bulk supplementation in 3d systems like organoids or mouse. Also, we think the bulk supplementation in iPS monoculture is akin to massive overexpression similar to

© 2024. Published by The Company of Biologists under the terms of the Creative Commons Attribution License (https://creativecommons.org/licenses/by/4.0/).

plasmids in 3t3 cells; we would think that means ceramide is being increased everywhere, so the local concentration in relevant organelles is being increased, but so is everything else. We have expanded and hopefully clarified our rationale on this in the revision.

-In Fig 1K, Smpd4 expression appears to be in granule cells, not PCs, is this correct?

Yes, this is correct. The text in the manuscript originally read as follows (p3), Smpd4 is expressed in multiple cell types of the cerebellum, most highly in granule cells but also in Calbindin-positive Purkinje cells (Fig. 1). As we revise, we will revisit to make sure the granule cell expression is not de-emphasized enough to be lost.

-In Fig. 2L,M the authors measure area but it seems that brain volume or weight would be better metrics for measuring brain/brain region size.

Brain weight is shown in Fig. 2E. Text reads Smpd4$^{null/null}$ animals had significantly smaller brains as measured by weight (Fig 2E) and dorsal surface area at P0 (n=4, p<0.001, Fig 2F,G,L) and P21 (Fig1 H-K).

-Fig. 3 – It would be helpful to show example images for all stainings (e.g. Pax6, PHH3, etc)

We have added a supplemental figure (Supp Fig 2) showing this data.

-In Fig. 3T and U, are Control and null mice statistically different with post-hoc testing? What statistical test was done here?

We have updated Fig. 3S,T,U to include all control genotypes to compare to the conditional mutant as we do elsewhere in the study. We included a preliminary version of this graph with more categories in the original submission which confused the issue.

-In Fig. 4B-D, DAPI or other nuclei stain should be shown to see the cell density and rosette architecture.

We have replaced these panels as suggested.

-Fig. 5E,F – is the ARL13B+ staining in magenta? If so, is it expected to stain the whole cell body? Are the “dots” of magenta the cilia? This should perhaps be verified with an additional cilia marker. From the example images is does not look like these “dots” are longer in the mutant tissue.

Also described in earlier comments, we have revised.

-For the inhibitor treatments in Fig. 6, is it possible that they are having a general non-specific effect on cell health, rather than regulating cilia production, specifically? For example, are treatments causing death of non-ciliated cells?

While this is certainly a formal possibility, it is not clear why that treatment would be restricted to, or preferential for, non-ciliated cells. We have reviewed the literature and, while ceramide treatment has been linked to cell death, we would suggest that these treatments would decrease cell death as they deplete ceramide.

Minor comments/suggestions:
-Is anything known about cilia from post-mortem patient specimens that would lend face validity to the findings reported here? (It's understood that these may not be available).

We inquired and there was no post-mortem analysis.

-It would be helpful to know what other brain regions Smpd4 is expressed in besides the cortex and cerebellum.

© 2024. Published by The Company of Biologists under the terms of the Creative Commons Attribution License (https://creativecommons.org/licenses/by/4.0/).

We have focused on the expression domains relevant to neurogenesis but expanded our presentation of the expression data and also discuss other resources suggesting Smpd4 is fairly widespread in the brain.

-It would be preferable to see the mRNA expression at 12.5, rather than stating data not shown.

We dramatically rearranged Fig1 with this comment partially in mind.

-Perhaps Fig. 1M should come first before discussing brain localization?

We dramatically rearranged Fig1 with this comment partially in mind.

-What about cerebellar development in the Smpd3-del mice? Perhaps the statement “dispensable for brain development” should be softened since other aspects of development could be altered, which were not looked at.

No Smpd3 KO homozygotes ever survived birth. We did look at embryonic cerebellum using sagittal sections of E14/16 Smpd3 homozygous knockout heads and didn’t notice any obvious cerebellar malformations. We did note that this stage is so early in cerebellar development that we probably wouldn’t see anything obvious anyway. However, the reviewer’s point is valid. We have softened this statement in this revision and the sentence now reads:
“Our data show that Smpd3 *is required for postnatal survival and skeletal development but loss of* Smpd3 *alone does not lead to any dramatic cortical malformations”*

-When describing Fig. 5A-D results, please indicate which mouse line the cultures were from.

We have clarified this issue in the revision.

-On page 7, the figure number is missing (it says “Fig I-M”)

This has been addressed in the revision (Fig. 5, I-M).

-On page 8 of the results, it would be helpful to indicate what GW4869 and FB1 are (although this is shown in the figure, it would be helpful to have it in the results as well).

Yes, this would be helpful and we have revised in this manner.

-Scale bars should be shown for all images in the supplementary figures (and also Fig. 2J, K).

Scale bars have been added in both the main and supplemental figures.

-It is unclear what the “Quit fraction” label indicates on Sup Fig. 2.

This has been more completely explained in the revised legend. This is now Supplemental Figure 1.

Second decision letter

MS ID#: DEVELOP/2023/202645

MS TITLE: SMPD4 mediated sphingolipid metabolism regulates brain and primary cilia development

AUTHORS: Katherine Inskeep, Bryan Crase, Thamara Dayarathna, and Rolf W Stottmann

I have now received the reports of two of the referees who had reviewed the first version of your manuscript, and I have reached a decision. The referees' comments are appended below, or you can access them online: please go to BenchPress and click on the 'Manuscripts with Decisions' queue in the Author Area.

© 2024. Published by The Company of Biologists under the terms of the Creative Commons Attribution License (https://creativecommons.org/licenses/by/4.0/).

The overall evaluation is positive and we would like to publish a revised manuscript in Development, provided that the referees' comments can be satisfactorily addressed. Please attend to all of the reviewers' comments in your revised manuscript and detail them in your point-by-point response. If you do not agree with any of their criticisms or suggestions explain clearly why this is so. If it would be helpful, you are welcome to contact us to discuss your revision in greater detail. Please send us a point-by-point response indicating your plans for addressing the referees' comments, and we will look over this and provide further guidance.

Reviewer 2

Advance summary and potential significance to field

In their manuscript, Inskeep et al. investigate the role of sphingolipid metabolism in brain development by analysing the function of the SMPD4 gene, mutations in which can lead to microcephaly and cerebellar hypoplasia. First, they showed that unlike human patients Smpd4 mutant mice did not develop a smaller brain and ruled out redundancy with another neutral sphingomyelinase, Smpd3, as a potential reason for the absence of a microcephaly phenotype. Smpd4 mutant mice though had a smaller cerebellum due to increased apoptosis of Purkinje neurons. In contrast, analysis of 2D neural cultures as well as 3D organoid models derived from either patient or CRISPR/Cas9 induced knock-out iPSCs revealed increased cell death of neural progenitors. Interestingly, primary cilia were longer and shorter in mouse Purkinje neurons and in human neural progenitors, respectively. Finally, ciliary length could be rescued by adding exogenous ceramide in the human models.

Taken together, the authors show that SMPD4 mediated generation of ceramide is critical for the development of primary cilia and the brain in humans and in mice. They use a valid combination of murine and human model systems to address the largely unexplored role of sphingolipid metabolism on brain development. Elucidating such functions is important given the severe consequences of SMPD4 mutations on human brain development, The manuscript is well written and the authors present convincing data, however, there are several shortcomings the authors need to address before publication.

Comments for the author

The authors have addressed most of the point I raised in my original review, but I am still sceptical about the human iPSC, 2D and organoid experiments. I do understand the technical difficulties with correcting the patient line but it should have been possible to generate a second, independent deletion line. As it stands, the authors used three technical replicates overall but only one biological replicate without any appropriate control for the patient line. Moreover, there is a huge difference in the number of differentially expressed genes between control deletion/mutant deletion lines on the one hand and control deletion/patient lines on the other hand. Taken together, these limitations raise severe doubts about the reproducibility of the data.

Minor point:

I agree that figures 5E/F have improved but it looks like the control cell has two cilia. A double staining with antibodies from two different species would help. There is an excellent mouse anti-ARL13B antibody available from Neuromab.

Reviewer 3

Advance summary and potential significance to field

The goal of this study is to investigate how SMPD4 (and Smpd3) contribute to early brain development. Mutations in SMPD4 cause a developmental disorder associated with microcephaly and other neurological alterations. The authors use mouse models and human cellular models to determine how mutations in SMPD4 affect the proliferation of progenitor cells and the survival of

© 2024. Published by The Company of Biologists under the terms of the Creative Commons Attribution License (https://creativecommons.org/licenses/by/4.0/).

newborn neurons. They also observe alterations in cilia production that can be reversed by supplementation with ceramides. The advances made by this study are the further characterizations of mouse lines with Smpd4 and Smpd3 mutations as well as the generation of human cellular models with SMPD4 mutations. Using these models, the authors show that the mutations impact both human and mouse brain development, albeit with some species specific effects. They also suggest that defective cilia may contribute to the developmental alterations observed in SMPD4 disorder.

Comments for the author

The changes made by the authors have addressed many of the comments and have improved by the paper. That said, I do still have some concerns regarding the overall conclusions that can be drawn from the study as stated below. For me it boils down to an editorial decision about whether or not these concerns preclude publication, as the data are generally rigorous and objectively reported in the study.

Specific points:
-While the authors have reported a lot of different observations that are likely of value to the field, it is still a bit difficult to put these into a conceptual framework. This is exemplified by Fig. 5 where cilia in different models are either unchanged, lengthened or shortened. In this case it's hard to understand how altered cilia are contributing to the disease phenotype(s) and which direction one would want to modify them therapeutically.
-I still find that ending on the RNAseq data is not particularly strong. The lack of overlap in DE genes between the KO and patient line is concerning and suggests that differences in the control vs. patient lines may be driven more by cell line artifacts rather than SMPD4 status. Indeed, the patient line seems to have acquired a chromosomal abnormality in culture, which is very likely to affect gene expression. Perhaps only the “isogenic” control and KO should be included in this analysis if the authors want to include it.
-In terms of the lack of PAX6 in the mutant organoids, the authors state that "
patient organoids containing virtually no NPCs (Fig 4V-Y)". The authors cannot conclude that there are no NPCs in these organoids without additional analysis as not all NPCs are PAX6-expressing. This is especially true for the Lancaster protocol, which produces cells of multiple brain lineages. The easy fix would be to just state that there is a reduction in PAX6+ cells without making any conclusions about the number or type of progenitor cells.

Minor comments:
-Page 3: Perhaps revise the first sentence of the second paragraph to “…assayed Smpd4 mRNA expression in the forebrain via RNAScope…”
-The first sentence of the discussion seems a bit overstated - "We have demonstrated that SMPD4 is critical for brain development in humans". Perhaps this could be revised to state that SMPD4 is critical for early neural development in stem cell-derived models
-For consistency, please include p values on graph for Fig. 4AF

Second revision

<u>Author response to reviewers' comments</u>

Reviewer 2 Advance Summary and Potential Significance to Field:
In their manuscript, Inskeep et al. investigate the role of sphingolipid metabolism in brain development by analysing the function of the SMPD4 gene, mutations in which can lead to microcephaly and cerebellar hypoplasia. First, they showed that unlike human patients Smpd4 mutant mice did not develop a smaller brain and ruled out redundancy with another neutral sphingomyelinase, Smpd3, as a potential reason for the absence of a microcephaly phenotype. Smpd4 mutant mice though had a smaller cerebellum due to increased apoptosis of Purkinje neurons. In contrast, analysis of 2D neural cultures as well as 3D organoid models derived from either patient or CRISPR/Cas9 induced knock-out iPSCs revealed increased cell death of neural progenitors. Interestingly, primary cilia were longer and shorter in mouse Purkinje neurons and

© 2024. Published by The Company of Biologists under the terms of the Creative Commons Attribution License (https://creativecommons.org/licenses/by/4.0/).

in human neural progenitors, respectively. Finally, ciliary length could be rescued by adding exogenous ceramide in the human models.

Taken together, the authors show that SMPD4 mediated generation of ceramide is critical for the development of primary cilia and the brain in humans and in mice. They use a valid combination of murine and human model systems to address the largely unexplored role of sphingolipid metabolism on brain development. Elucidating such functions is important given the severe consequences of SMPD4 mutations on human brain development, The manuscript is well written and the authors present convincing data, however, there are several shortcomings the authors need to address before publication.

Reviewer 2 Comments for the Author:
The authors have addressed most of the point I raised in my original review, but I am still sceptical about the human iPSC, 2D and organoid experiments. I do understand the technical difficulties with correcting the patient line but it should have been possible to generate a second, independent deletion line. As it stands, the authors used three technical replicates overall but only one biological replicate without any appropriate control for the patient line. Moreover, there is a huge difference in the number of differentially expressed genes between control deletion/mutant deletion lines on the one hand and control deletion/patient lines on the other hand. Taken together, these limitations raise severe doubts about the reproducibility of the data.

We understand this (new) concern of the reviewer. We agree this generation of an independent deletion clone would have been ideal and submit that the “gold standards” for this kind of work are an evolving set of ideas that may not have fully matured and been established within the community when we initially undertook these stem cell studies.

We do note and discuss the larger number of differentially expressed genes between the patient line and the others and offer some thoughts on why this may be in the manuscript. We have also added a further discussion point about these limitations.

Minor point:

I agree that figures 5E/F have improved but it looks like the control cell has two cilia. A double staining with antibodies from two different species would help. There is an excellent mouse anti-ARL13B antibody available from Neuromab.

We have repeated this studies with the new antibody recommended by the reviewer and present this in a revised Figure 5. These results are consistent with the previous data.

*****
Reviewer 3 Advance Summary and Potential Significance to Field:
The goal of this study is to investigate how SMPD4 (and Smpd3) contribute to early brain development. Mutations in SMPD4 cause a developmental disorder associated with microcephaly and other neurological alterations. The authors use mouse models and human cellular models to determine how mutations in SMPD4 affect the proliferation of progenitor cells and the survival of newborn neurons. They also observe alterations in cilia production that can be reversed by supplementation with ceramides. The advances made by this study are the further characterizations of mouse lines with Smpd4 and Smpd3 mutations as well as the generation of human cellular models with SMPD4 mutations. Using these models, the authors show that the mutations impact both human and mouse brain development, albeit with some species specific effects. They also suggest that defective cilia may contribute to the developmental alterations observed in SMPD4 disorder.

Reviewer 3 Comments for the Author:
The changes made by the authors have addressed many of the comments and have improved by the paper. That said, I do still have some concerns regarding the overall conclusions that can be drawn from the study as stated below. For me it boils down to an editorial decision about whether or not these concerns preclude publication, as the data are generally rigorous and objectively reported in the study.
Specific points:
-While the authors have reported a lot of different observations that are likely of value to the field,

© 2024. Published by The Company of Biologists under the terms of the Creative Commons Attribution License (https://creativecommons.org/licenses/by/4.0/).

it is still a bit difficult to put these into a conceptual framework. This is exemplified by Fig. 5 where cilia in different models are either unchanged, lengthened or shortened. In this case it's hard to understand how altered cilia are contributing to the disease phenotype(s) and which direction one would want to modify them therapeutically.

We agree this is challenging to put into one satisfactory model to explain all the data. These are the findings we are prepared to offer at this time. We would note that the data about cilia length are different between models (human cells and mouse tissue) but are consistent within the model system. We have commented on this previously in the Discussion and now also mention in a new section of the Discussion on limitations to the study.

-I still find that ending on the RNAseq data is not particularly strong. The lack of overlap in DE genes between the KO and patient line is concerning and suggests that differences in the control vs. patient lines may be driven more by cell line artifacts rather than SMPD4 status. Indeed, the patient line seems to have acquired a chromosomal abnormality in culture, which is very likely to affect gene expression. Perhaps only the “isogenic” control and KO should be included in this analysis if the authors want to include it.

We understand this reservation from the reviewer and have discussed this with the editorial staff.

-In terms of the lack of PAX6 in the mutant organoids, the authors state that " patient organoids containing virtually no NPCs (Fig 4V-Y)". The authors cannot conclude that there are no NPCs in these organoids without additional analysis as not all NPCs are PAX6-expressing. This is especially true for the Lancaster protocol, which produces cells of multiple brain lineages. The easy fix would be to just state that there is a reduction in PAX6+ cells without making any conclusions about the number or type of progenitor cells.

We have made this edit.

Minor comments:
-Page 3: Perhaps revise the first sentence of the second paragraph to “...assayed Smpd4 mRNA expression in the forebrain via RNAScope...”

We have made this edit.

-The first sentence of the discussion seems a bit overstated - "We have demonstrated that SMPD4 is critical for brain development in humans". Perhaps this could be revised to state that SMPD4 is critical for early neural development in stem cell-derived models

We have made this edit.

-For consistency, please include p values on graph for Fig. 4AF

We have made this edit.

<u>Third decision letter</u>

MS ID#: DEVELOP/2023/202645

MS TITLE: SMPD4 mediated sphingolipid metabolism regulates brain and primary cilia development

AUTHORS: Katherine Inskeep, Bryan Crase, Thamara Dayarathna, and Rolf W Stottmann

I am delighted to tell you that your manuscript has been accepted for publication in Development, pending our standard publication integrity checks.

© 2024. Published by The Company of Biologists under the terms of the Creative Commons Attribution License (https://creativecommons.org/licenses/by/4.0/).
